# Closing the Cathode Loop in Lithium-Ion Battery Recycling: From Metal Recovery to Direct Regeneration of NMC and LFP Cathodes

**DOI:** 10.3390/ma19214753

**Published:** 2026-11-09

**Authors:** Arman Umirzakov, Yedil Otunchi, Alimzhan Serikbekov, Ainagul Kemelbekova, Aigul Shongalova, Ekaterina Bondar, Elena Dmitriyeva

**Affiliations:** 1 Institute of Physics and Technology, Satbayev University, Ibraimov Str., 11, Almaty 050013, Kazakhstan; arman.umirzakov.89@gmail.com (A.U.); eotynshy@gmail.com (Y.O.); alimzhan.serikbekov2002@gmail.com (A.S.); shongalova.aigul@gmail.com (A.S.); katebondar111@gmail.com (E.B.); y.dmitriyeva@satbayev.university (E.D.); 2 Department of Materials Science, Nanotechnology and Engineering Physics, Satbayev University, Satbayev Str., 22a, Almaty 050013, Kazakhstan

**Keywords:** lithium-ion battery recycling, direct regeneration, relithiation, LiFePO_4_, Ni-rich cathodes, hydrometallurgy, black mass, graphite recycling, life-cycle assessment, circular economy

## Abstract

Lithium-ion battery (LIB) recycling is usually compared across pyrometallurgical, hydrometallurgical and direct routes as if the optimal route were independent of the cathode being recycled. This review argues that cathode chemistry is the governing variable. Following the chain from cathode chemistry and degradation mechanism through pretreatment, recycling route and recovered product to electrochemical validation, we compare LiNi_x_Mn_y_Co_z_O_2_ (NMC, 111 to 811) and LiFePO_4_ (LFP), with LiCoO_2_ and LiNi_0.8_Co_0.15_Al_0.05_O_2_ as references. LFP degradation is dominated by Li loss and Fe/Li anti-site defects in an intact olivine framework, which makes direct regeneration mechanistically well founded. Ni-rich NMC suffers microcracking, surface reconstruction and proton sensitivity, so its regeneration increasingly becomes upcycling into a different product, whereas hydrometallurgical resynthesis currently has the strongest industrial validation. Reported recovery efficiencies above 95% rarely demonstrate battery-grade products, and published energy estimates for the same route and chemistry differ by a factor of up to about 1.5 because of inconsistent system boundaries. We propose a cathode-to-cathode circularity framework and a minimum reporting set that link material recovery to validation in new cells.

## 1. Introduction

The chemistry of the batteries that will reach end of life in the next decade differs sharply from the chemistry for which today’s recycling industry was designed. LiFePO_4_ (LFP) supplied almost half of the global electric-car market in 2024, compared with less than 10% in 2020 [1]. In 2025 LFP accounted for more than half of electric-vehicle (EV) batteries and more than 90% of stationary storage batteries, and LFP packs were on average more than 40% cheaper per kWh than LiNi_x_Mn_y_Co_z_O_2_ (NMC) packs [2]. At the same time, almost 80% of EV batteries deployed outside China in 2025 used Ni-containing chemistries, and high-Ni chemistries rely on relatively small quantities of Co while LFP contains none; together they account for the vast majority of the EV battery market [3]. Recyclers will therefore receive a feed that is simultaneously lower in cobalt, more heterogeneous, and, for a growing share, free of Ni and Co altogether.

This matters because the economics of established routes were built on metal value. The Umicore-type smelting process recovers Co, Ni and Cu as an alloy while Li reports to the slag, and its economic efficiency depends on the cobalt price and cobalt content of the feed [4]. An early supply-chain analysis excluded LFP from consideration on the expectation that layered cathodes would displace it in vehicles [5]; market development has reversed that expectation. Life-cycle work also showed early that recycling LFP by pyro- or hydrometallurgy does not yield net avoided greenhouse-gas emissions, whereas direct cathode recycling can reduce emissions for Ni- and Co-containing cathodes [6].

**Figure 1:**
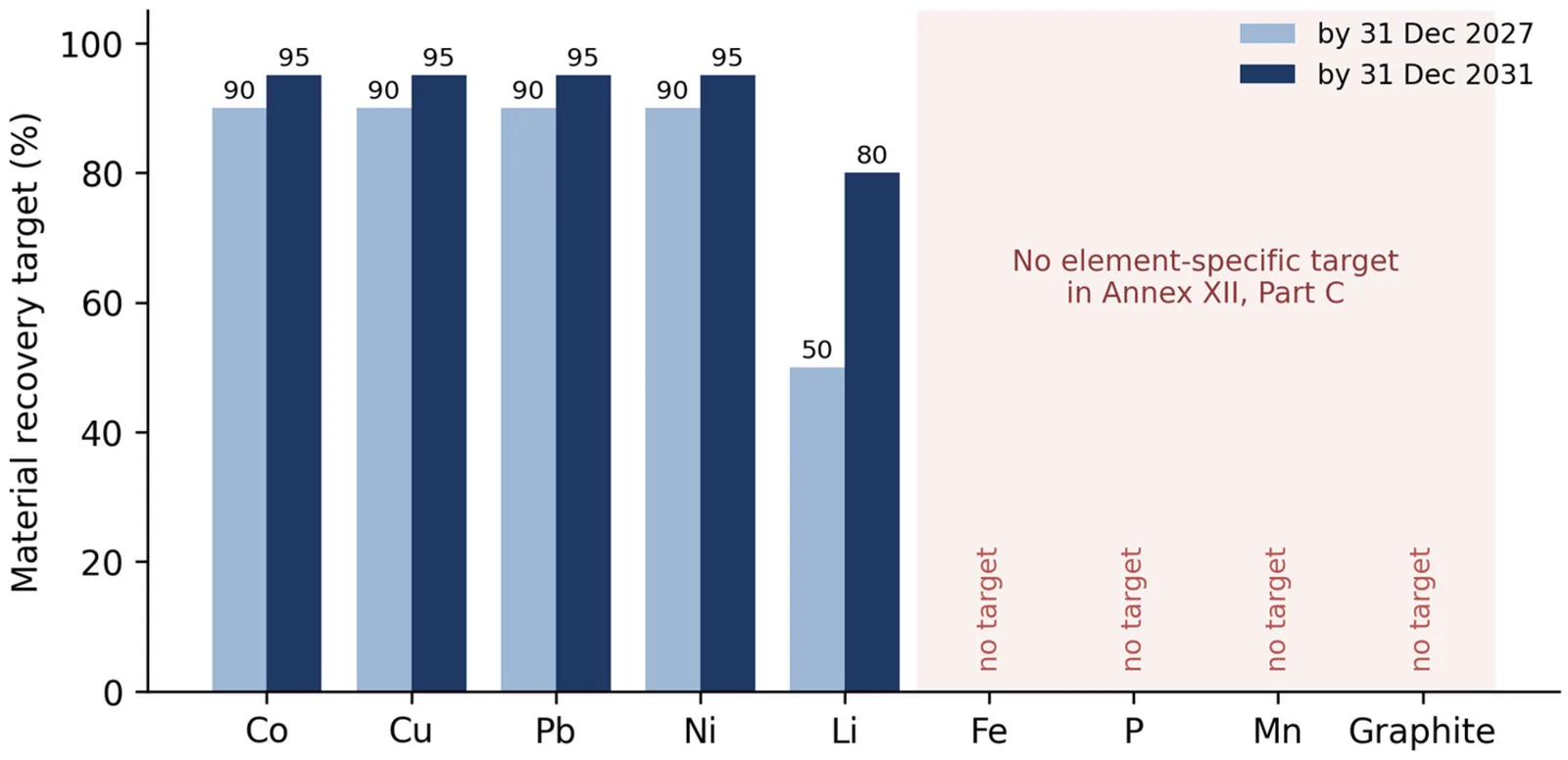
Material recovery targets for waste batteries of Regulation (EU) 2023/1542 [7]; Fe, P, Mn and graphite have no element-specific recovery target.

Regulation is tightening in parallel, but it is itself metal-centred. Under Regulation (EU) 2023/1542, recyclers must recover 90% of Co, Cu, Ni and Pb and 50% of Li by the end of 2027, rising to 95% and 80% by the end of 2031; recycling efficiency for Li-based batteries must reach 65% by weight by 2025 and 70% by 2030; and minimum recycled contents of 16% Co, 6% Li and 6% Ni apply from August 2031 [7] (Figure 1). No element-specific recovery targets exist for Fe, P, Mn or graphite. Because Fe, P and graphite make up most of the active mass of an LFP cell, a recycler can comply with the metal-recovery targets while destroying the functional value of an LFP cathode and still earn little revenue from the operation.

The practical consequences of this metal-centred metric are most acute for LFP. First, the only cathode element of an LFP cell that carries a material-recovery target is Li, so compliance can be demonstrated by recovering Li (and the Cu of the current collectors) alone, for example by selective leaching, while FePO_4_, iron oxides or phosphate-bearing residues are landfilled or sold as low-grade by-products. Second, the recycling-efficiency target is weight-based (65% by 2025 and 70% by 2030 [7]) and counts all recovered materials equally, so casings, Al and Cu foils and other non-functional fractions can make a large contribution to compliance even when the functional cathode is destroyed; the metric does not distinguish between recovering a cathode and recovering its housing. Third, because no requirements are set for Fe, P, Mn, Al, F or graphite, these elements are still treated in industrial practice as impurities rather than products [8], and graphite is commonly consumed as a reductant or fuel in thermal processes [4]. Fourth, the recycled-content obligations that apply from 2031 cover only Co, Li, Ni and Pb [7], so there is no regulatory demand for recycled Fe, P or graphite. As a result, the revenue of metal-centred LFP recycling depends almost entirely on the Li price, which makes it economically fragile when Li prices fall, and the regulation provides no direct incentive for value-preserving routes such as direct regeneration, which returns Fe, P, Li and the carbon coating together in a functional form. A metric that rewards the recovery of functional cathode material rather than of individual elements would align regulatory compliance with the circularity framework proposed in Section 7.

**Figure 2:**
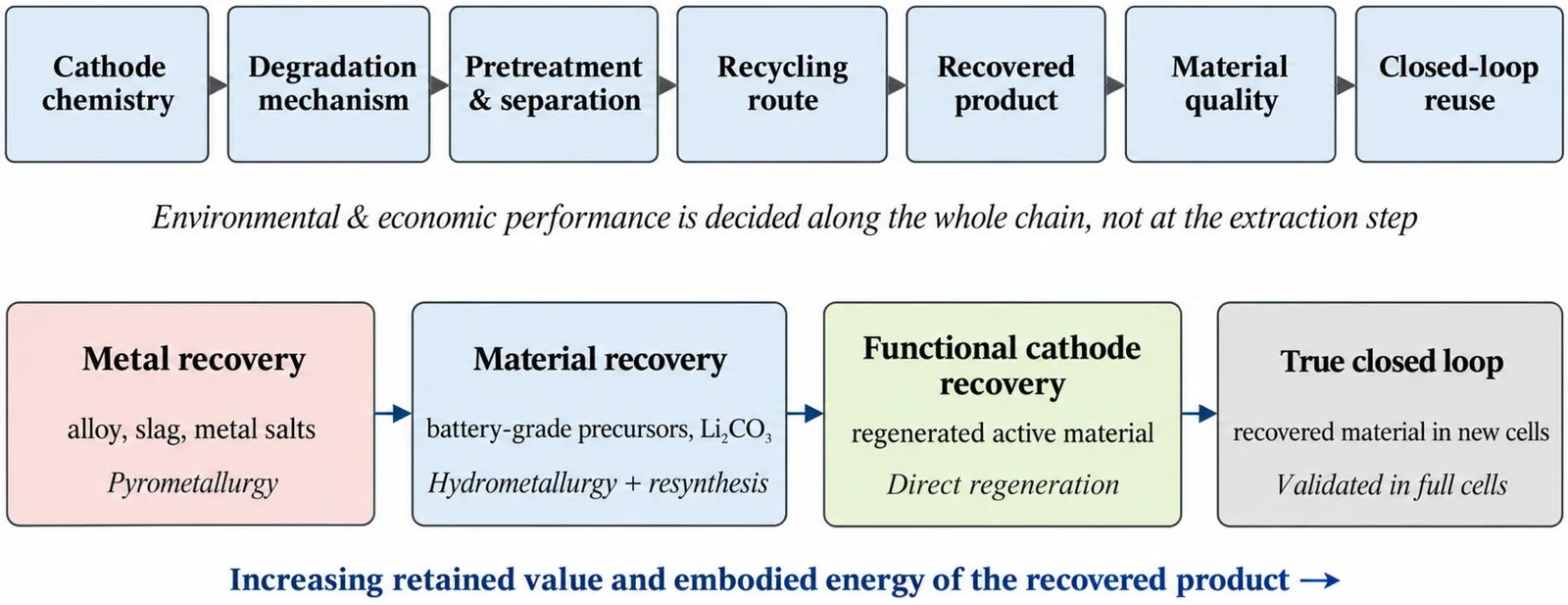
Conceptual framework of this review: the cathode-centred chain from chemistry to closed-loop reuse (**top**) and the four levels of recovered product, from metal recovery to a validated closed loop (**bottom**).

Many comprehensive reviews compare pyrometallurgy, hydrometallurgy and direct recycling [4,9,10,11,12]. Most are organised around process routes and treat cathode chemistry as a parameter that modifies route performance. They also commonly rank processes by elemental extraction efficiency, although a leach yield of 99% does not establish that a battery-grade precursor or a functional cathode has been produced. The distinct contribution of this review is therefore threefold: it uses cathode chemistry and degradation mode to select the recycling route; it separates metal recovery, precursor recovery, functional-material recovery and validation in a new cell; and it proposes a six-gate cathode-to-cathode framework with a minimum reporting set. We apply this approach to NMC and LFP, using LiCoO_2_ (LCO) and LiNi_0.8_Co_0.15_Al_0.05_O_2_ (NCA) as reference chemistries, and explicitly distinguish laboratory, pilot-scale and commercial evidence. Figure 2 presents the overall logic of the cathode recycling pathway, showing how the final environmental and economic performance is determined by the entire sequence of processing steps rather than by metal extraction alone. The pathway begins with the cathode chemistry and its degradation mechanism, which define the condition of the spent material and influence the choice of pretreatment and separation strategy. The quality of the separated feed then determines which recycling route can be applied and what type of product can ultimately be recovered. Conventional pyrometallurgical processing mainly leads to metal recovery in the form of alloys, slags, or metal salts, whereas hydrometallurgical routes can retain a greater fraction of the material value by producing battery-grade precursors that are subsequently resynthesised into cathode materials. Direct regeneration preserves an even higher level of embodied value by restoring the spent cathode material itself rather than decomposing it completely into individual elements. The highest degree of circularity is achieved only when the recovered material reaches the required quality, is electrochemically validated, and is successfully reused in new battery cells. Accordingly, the recycling process should be evaluated as a continuous chain from cathode chemistry and degradation state through pretreatment, recovery, product quality, and final reuse, because each stage affects the amount of functional and embodied value retained in the recovered product.

### 1.1. Scope and Literature Search Strategy

This article is a critical (narrative) review rather than a systematic review or meta-analysis, but its literature base was assembled with a defined search so that statements about the absence of evidence can be interpreted. Scopus, Web of Science Core Collection and Google Scholar were searched for publications from January 2005 to September 2026 using combinations of the terms (“lithium-ion batter*” OR “Li-ion batter*”) AND (recycl* OR regenerat* OR relithiat* OR upcycl* OR “black mass”) AND (cathode OR NMC OR NCM OR LFP OR LiFePO_4_ OR LiCoO_2_ OR NCA OR graphite). These were complemented by targeted searches on flotation, binder removal, fluorine impurities, life-cycle assessment and techno-economic analysis, and by backward and forward citation tracking of key papers. Peer-reviewed journal articles in English, together with regulations and reports of international agencies, were considered. Studies were retained if they reported (i) primary quantitative data on the degradation, pretreatment, leaching, regeneration, upcycling or resynthesis of LCO, NMC, NCA, LFP or graphite from spent batteries or production scrap, or (ii) primary life-cycle or techno-economic results for recycling routes. Studies on pristine (never-cycled) materials without a recycling context, patents and non-peer-reviewed sources other than regulatory and agency documents were excluded. Reviews were used for context but, whenever the primary study was accessible, not as a source of quantitative values. Statements in this review that a topic was not found refer to this search and should be read as “to the authors’ knowledge”.

### 1.2. Terminology

Three levels of value-preserving recovery are distinguished throughout this review, and the terms are used only in the following sense. Regeneration denotes the restoration of the spent active material toward its original composition, crystal structure and particle morphology without dissolution, typically by relithiation followed by annealing. Upcycling denotes a non-dissolving route in which the composition, dopant content or particle morphology of the spent material is deliberately changed to yield a different cathode product, for example the conversion of polycrystalline NMC811 into single-crystal NMC622. Resynthesis denotes the dissolution of the spent cathode by hydrometallurgical (wet-chemical) processing, recovery of a precursor and re-sintering into a new cathode material. “Direct recycling” is used as an umbrella term for regeneration and upcycling, and “metal recovery” for routes whose products are alloys, salts or metal compounds that are not themselves cathode precursors.

## 2. Cathode Chemistry as the Governing Variable: Composition, Value and Degradation

### 2.1. Composition and Recoverable Value

Cathode chemistry is one of the key factors determining both the technical feasibility and the economic attractiveness of lithium-ion battery recycling. The elemental composition of the cathode defines which materials can be recovered, their potential market value, and the extent to which conventional metal-centred recycling remains economically justified. At the same time, the intrinsic value of a spent cathode is not limited to the recoverable metals alone. Its crystal structure, particle morphology, electrochemical functionality, and degree of structural degradation can also represent a significant retained value, particularly when direct regeneration is considered. Therefore, a meaningful assessment of recycling potential requires consideration of both the elemental composition of the cathode and the functional value preserved in the spent material.

These differences become particularly important as the battery market shifts from Co-rich cathodes toward Ni-rich NMC compositions and Co-free chemistries such as LFP. In such systems, the relative contribution of high-value transition metals changes substantially, which directly affects the economic logic of recycling. Consequently, the preferred recycling strategy may differ not only between layered oxides and olivine-type cathodes, but also among different NMC compositions with varying Ni, Co, and Mn contents.

**Figure 3:**
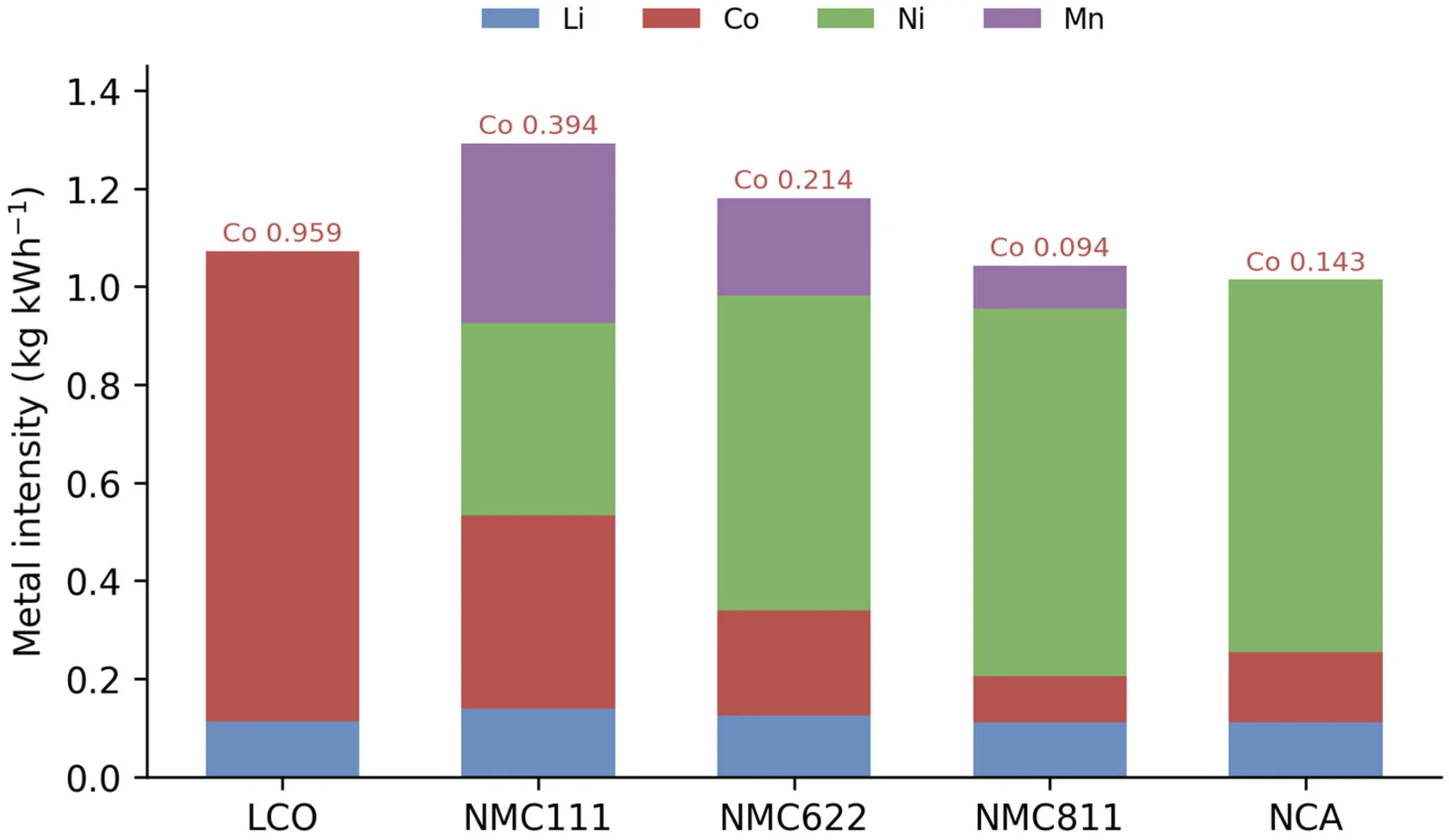
Metal intensity of layered-oxide cathodes (kg of metal per kWh of cell energy). Figure created by the authors using data from Olivetti et al. [5]; LFP is omitted because Li is its only constituent of significant commodity value.

**Figure 4:**
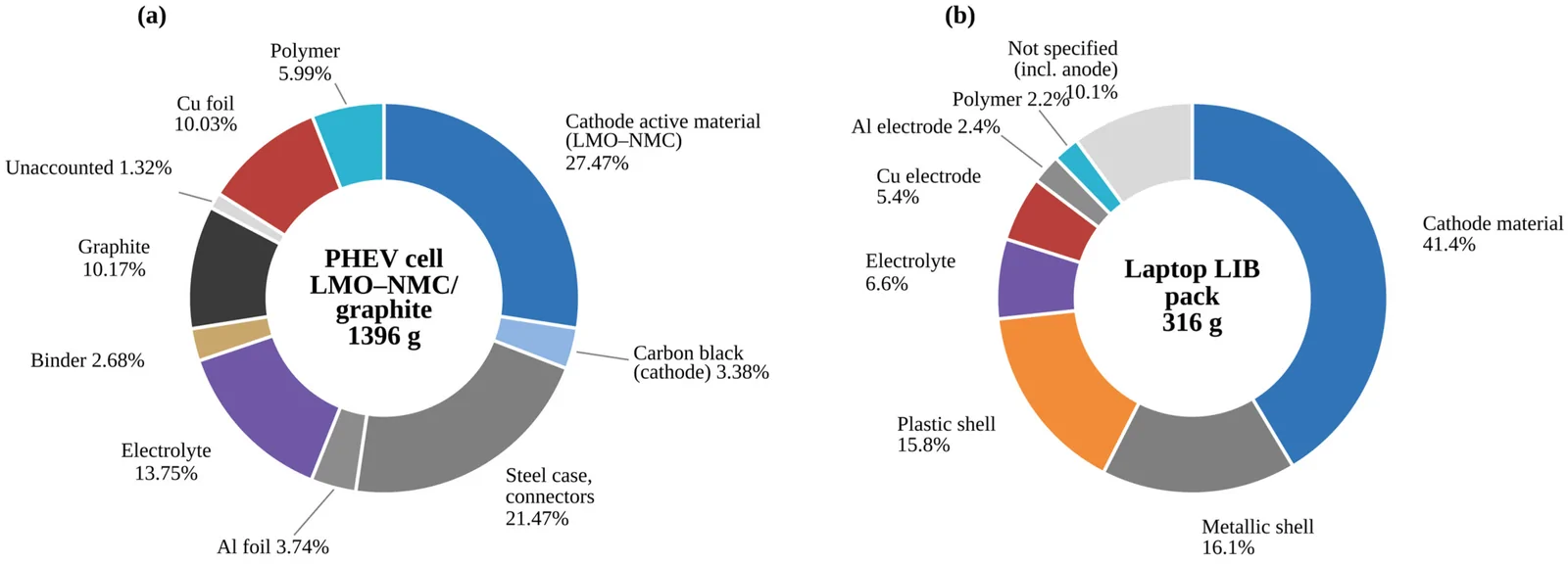
Mass composition of (**a**) a 1396 g LiMn_2_O_4_–NMC/graphite traction cell for a plug-in hybrid electric vehicle, determined by dismantling, and (**b**) a 316 g laptop lithium-ion battery pack. Figure created by the authors using data from Cusenza et al. [17] (**a**) and from [18] (**b**).

Figure 3 and Figure 4 illustrate two complementary aspects that determine the recycling value of lithium-ion batteries: the elemental composition of the cathode and the distribution of materials at the pack level. Figure 3 compares the metal intensity of the principal layered-oxide cathode families and clearly demonstrates how the transition toward Ni-rich chemistries changes the economic basis of recycling. In particular, moving from NMC111 to NMC622 and NMC811 progressively decreases the Co content while increasing the fraction of Ni. The Co intensity decreases from 0.394 kg kWh^−1^ in NMC111 to 0.214 kg kWh^−1^ in NMC622 and only 0.094 kg kWh^−1^ in NMC811, whereas the Ni content increases from 0.392 to 0.641 and 0.750 kg kWh^−1^, respectively [5]. LCO represents the opposite extreme, containing approximately 0.959 kg kWh^−1^ of Co and therefore historically offering a relatively high intrinsic metal value to recycling processes [5]. NCA also contains a comparatively low amount of Co, about 0.143 kg kWh^−1^, while its metal inventory is dominated by Ni. Thus, the gradual reduction of Co loading in modern cathodes lowers the contribution of one of the traditionally most valuable recovered metals and shifts the economic importance toward Ni and Li.

At the same time, the Li intensity varies much less strongly among layered-oxide cathodes, remaining within a relatively narrow range of approximately 0.11–0.14 kg kWh^−1^ [5]. This has an important implication for emerging battery chemistries. In LFP, for example, Li is essentially the only constituent with substantial commodity value, whereas Fe, P, and O contribute comparatively little to the recoverable elemental value [13,14]. Consequently, recycling strategies that focus exclusively on the recovery of individual metals may become less attractive for low-Co or Co-free cathodes.

This effect is reinforced by the evolution of cathode performance. Practical specific capacity increases with increasing Ni content, from about 140 mAh g^−1^ for LCO to approximately 160, 180, and 200 mAh g^−1^ for NMC111, NMC622, and NMC811, respectively [15]. LFP provides a practical specific capacity of approximately 160 mAh g^−1^ at an operating voltage of about 3.5 V [16]. Therefore, in chemistries such as LFP, a considerable fraction of the value of the spent cathode is associated not simply with the market price of its constituent elements, but with the preserved crystal structure, particle morphology, electrochemical functionality, and the possibility of restoring the material for reuse.

Figure 4 further emphasizes the importance of the cathode from a whole-cell and pack perspective. Because the market, regulatory and economic framing of this review is centred on EV and stationary-storage batteries, Figure 4a shows the bill of materials of an automotive cell obtained by dismantling: in a 1396 g LiMn_2_O_4_–NMC/graphite prismatic cell for a plug-in hybrid electric vehicle, the cathode active material is the largest single fraction at 27.5 wt% (30.9 wt% including the carbon black of the cathode coating), followed by the steel case and connectors (21.5 wt%), the electrolyte (13.8 wt%), graphite (10.2 wt%) and Cu foil (10.0 wt%) [17]. At the level of a complete EV pack, module and pack housings, thermal management and electronics dilute this share further. The consumer pack in Figure 4b is retained for comparison because it provides a complete pack-level mass balance including the casing. In the 316 g laptop LIB pack (Figure 4b), the cathode material constitutes the largest individual mass fraction, accounting for 41.4% of the total pack mass [18]. This fraction is substantially greater than that of the metallic shell (16.1%) and the plastic shell (15.8%), while the electrolyte represents 6.6%, the Cu electrode 5.4%, the Al electrode 2.4%, and polymeric components 2.2%. A further 10.1% is classified as unspecified material, including contributions associated with other cell components [18]. Although the precise mass balance depends on cell format, chemistry, design, and application, both examples demonstrate that the cathode is not a minor component of the battery system but one of its dominant material reservoirs.

The comparison of Figure 3 and Figure 4 therefore highlights an important shift in the logic of lithium-ion battery recycling. Although the cathode remains one of the most significant material components of the cell, the economic and strategic value of its recovery increasingly depends on cathode chemistry. In Co-rich systems such as LCO, conventional metal recovery can still capture a substantial share of the material value. By contrast, for Ni-rich NMC, NCA, and particularly LFP, greater benefit may be obtained by preserving the material in a form that can be returned more directly to cathode production. This favors a transition from purely extraction-oriented processes toward precursor recovery, resynthesis, and direct regeneration, where the objective is not only elemental recovery but also retention of the processing effort and functional characteristics already embodied in the cathode material.

However, the suitability of a cathode for such value-preserving routes depends not only on its initial composition but also on the nature and extent of degradation accumulated during service. Loss of cyclable lithium, cation disorder, surface reconstruction, particle cracking, interfacial reactions, and structural defects can affect whether the original material can be restored directly or must instead be decomposed and resynthesised. The degradation mechanism is therefore a second governing variable, alongside cathode chemistry, in determining the most appropriate recycling strategy.

### 2.2. What Happens to Cathodes During Ageing

Layered oxides. Degradation of NMC combines loss of cyclable Li, Li^+^/Ni^2+^ cation mixing, formation of a surface rock-salt reconstruction layer associated with oxygen loss and transition-metal reduction, and the growth of a cathode–electrolyte interphase from electrolyte decomposition products [15]. For Ni-rich compositions a mechanical mode dominates. Ryu et al. showed that for x > 0.8 in Li[Ni_x_Co_y_Mn_1 − x − y_]O_2_, a phase transition near the end of charge causes abrupt anisotropic lattice contraction. The resulting residual stress lets internal microcracks propagate to the particle surface, where they open channels for electrolyte penetration and degradation of newly exposed internal surfaces [19]. Microcracks also develop during calendar ageing, the rock-salt passive layer near cracks exfoliates, and this microstructural damage worsens with increasing Ni content [20]. In recycling-derived NMC811 examined after service, He et al. measured a Li/(Ni+Co+Mn) ratio of 0.73, a 5.15% NiO rock-salt phase and 6.5% Li/Ni mixing, together with fragmented secondary particles [21].

Olivine LFP. In LFP the framework is robust and degradation is mainly compositional. After up to 6500 cycles and up to 50% capacity decay, Xu et al. found 47.1% Li deficiency and 4.81% Fe/Li anti-site defects, while particle morphology and the 2–3 nm carbon shell were preserved [14]. Ji et al. reported 21.3 wt% FePO_4_ and 2.4% anti-site defects in spent 18,650 cells, with degradation concentrated at particle surfaces and partial loss of the carbon coating [13].

### 2.3. Reversible Versus Irreversible Degradation: The Scientific Basis for Choosing a Route

The choice between direct regeneration and more destructive recycling routes depends strongly on whether the dominant degradation mechanisms can be reversed without completely rebuilding the cathode material. From this perspective, degradation should be considered not only in terms of capacity loss, but also in terms of the type of structural and chemical changes that have accumulated during service. Some defects primarily involve lithium inventory, oxidation state, or local cation disorder and can potentially be corrected by relithiation and moderate thermal treatment. Other forms of degradation involve extensive mechanical damage, transition-metal loss, or deep structural reconstruction and therefore require more intensive processing. Distinguishing between these two categories provides a practical basis for assessing whether the original cathode structure can be preserved or whether the material must be dissolved and resynthesised.

Table 1 classifies the degradation modes discussed above according to whether relithiation and short annealing can reverse them. Li loss, Fe(III) formation, Fe/Li anti-site defects and thin surface rock-salt layers are reversible: relithiation in solution or in a Li-containing melt, with a reductant where Fe^3+^ is involved, followed by short annealing, has restored composition and structure in both NMC523 and LFP [14,22]. Microcrack networks, particle fragmentation, transition-metal dissolution and deep bulk disorder are not reversed by relithiation. They can be removed only by recrystallisation, which changes particle morphology, for example into single crystals, or by dissolution.

Two further aspects determine whether the reversible modes can be exploited in practice. The first is the spatial distribution of damage. In LFP, degradation is concentrated at particle surfaces [13], so short, low-temperature treatments can reach the damaged regions more readily. In Ni-rich NMC, crack networks can extend through secondary particles [19], and effective repair requires mass transport over a much larger volume, which generally increases the required treatment temperature and duration. The second is heterogeneity within the feed. Cells reach end of life at different states of health, so any process that relies on stoichiometric lithium addition requires prior estimation of the lithium deficiency. Solution relithiation is attractive in this respect because it can be self-limiting, as demonstrated for LFP cells with 40%, 50% and 85% state of health [14].

It should also be recognised that most quantitative degradation data are derived from laboratory-cycled cells, whereas field-aged batteries experience a combination of cycling and calendar ageing. This distinction is important because calendar ageing can introduce additional microstructural damage, including microcracking in Ni-rich cathodes [20], which may reduce the effectiveness of otherwise reversible regeneration treatments.

For route selection this has three practical consequences. First, the entries marked as reversible in Table 1 should be regarded as conditional for field-aged cells: accelerated calendar ageing in the charged state produces microcracks in Ni-rich NMC [20], so a nominally reversible Li-inventory loss may be accompanied by damage that relithiation does not repair, and the cell then requires recrystallisation rather than simple regeneration. Second, cracks formed during cycling or storage are penetrated by electrolyte [19], so field-aged material can contain electrolyte-derived species such as LiF and fluorophosphates inside secondary particles rather than only on their outer surfaces; such species are not removed by surface washing and can be incorporated into the lattice during annealing [23]. Third, in LFP/graphite cells from long storage or stationary service, much of the capacity loss generally reflects Li consumed in the anode SEI, which appears in the cathode as Li deficiency; this favours relithiation but makes the required Li dose dependent on the history of each cell. Because the evidence in Table 1 comes almost entirely from laboratory-cycled cells, regeneration studies should report the ageing history of the feed (cycling, calendar or field ageing, and storage time and temperature where known) and include at least one field-aged or calendar-aged sample before a “Yes” entry is used for route selection.

## 3. Pretreatment and Black Mass: Where Cathode Quality Is Decided

Pretreatment is a critical stage in lithium-ion battery recycling because it determines the composition, purity, and physical form of the material entering the subsequent recovery process. Regardless of whether the final route is pyrometallurgical, hydrometallurgical, or based on direct cathode regeneration, spent batteries must first be converted from intact cells or modules into processable material streams. These streams typically include metallic casing, current collectors, polymers, electrolyte-derived residues, and a fine electrode-powder fraction commonly referred to as black mass. The quality required of this black mass depends on the downstream route and is discussed for each pretreatment step below and summarised in Table 2.

Pretreatment should consequently be viewed not as a simple preparatory operation, but as a sequence of interdependent steps in which safety, particle liberation, binder removal, thermal history, and phase separation all influence the quality of the recovered cathode fraction. Discharge and dismantling reduce operational hazards and expose the internal cell components; mechanical comminution releases the electrode materials from the casing and current collectors; binder-removal treatments increase particle liberation; and subsequent physical separation is used to enrich the cathode fraction and remove graphite and metallic impurities. The effectiveness of each step therefore determines how much of the original functional material can be preserved for further processing.

### 3.1. Discharge, Dismantling and Comminution

The first challenge in battery pretreatment is safe handling of residual electrical energy. Residual charge creates a risk of short circuit, thermal runaway, fire, and explosion during crushing or shredding, which is why cells are commonly discharged before mechanical processing [4]. The degree of discharge and the selected dismantling strategy depend on battery format, state of charge, and the subsequent treatment route.

An alternative approach is to incorporate residual energy directly into a controlled thermal pretreatment. Industrial reductive calcination, for example, can process energised batteries with different states of charge and cell formats without a separate complete-discharge step. At Redwood Materials, this operation has been reported to account for only 5.5–7.5% of the environmental impact of the subsequent refining process [24]. This demonstrates that industrial flowsheets can integrate safety management and material conditioning within the same processing stage.

After electrical hazards have been controlled, mechanical operations are used to liberate the internal battery components. Crushing, shredding, sieving, and classification can separate large fractions such as metallic casing and current-collector foils from the finer electrode material [16]. Early mechanical flowsheets showed that crushing, sieving, and elutriation can produce distinct streams of foils, casing, and electrode powder [16]. However, complete liberation is difficult because the electrode structure is highly interpenetrated. Cathode and anode particles are attached to metallic foils through polymer binders and are mixed with conductive carbon and electrolyte residues. As a result, the black mass obtained after mechanical treatment is not a pure cathode product but a complex mixture whose composition depends strongly on the preceding dismantling and comminution conditions.

This limitation becomes particularly important for direct recycling. In hydrometallurgy, many residual components can later be dissolved, precipitated, or separated chemically. In direct regeneration, by contrast, impurities that remain after mechanical pretreatment can persist through the subsequent relithiation and annealing steps. Therefore, comminution must achieve sufficient liberation without introducing excessive contamination or damaging the active particles beyond the level that can subsequently be repaired.

### 3.2. Binder Removal

One of the main barriers to efficient separation of electrode materials is the polyvinylidene fluoride (PVDF) binder. PVDF bonds the active material to the current collectors and also coats particle surfaces, reducing the efficiency of subsequent physical separation. Binder removal is therefore an essential step when the objective is to recover a sufficiently clean cathode fraction.

Solvent-based removal with N-methyl-2-pyrrolidone (NMP) is effective because it dissolves PVDF and facilitates detachment of the active material from the foil [16]. This approach has also been used in several direct-regeneration studies to harvest relatively clean cathode powders [14]. Its main limitations are solvent cost, toxicity, solvent recovery requirements, and the difficulty of transferring laboratory-scale washing procedures directly to large industrial streams.

Thermal treatment provides a simpler alternative. Calcination at approximately 500 °C for several hours can decompose PVDF and remove part of the conductive carbon [21]. This improves particle liberation but also introduces a new problem: fluorine-containing species generated from the binder and electrolyte may remain in the solid residue. In spent NMC622 powder, compounds such as AlF3, LiF, and LixPFyO4 have been detected after thermal pretreatment and can subsequently be incorporated into regenerated materials [23]. Thus, binder removal cannot be evaluated only by how efficiently it cleans particle surfaces; the chemical form and final location of fluorine must also be considered.

The allowable pretreatment temperature is also chemistry-dependent. When industrial NMC523 black mass was roasted in air, NiO rock-salt reflections appeared at 600 °C, whereas the layered structure remained preserved at 525–550 °C [25]. The lower temperature range was therefore selected to avoid introducing structural degradation during pretreatment itself. This is especially important for direct regeneration, because excessive thermal exposure can generate the same disordered or rock-salt surface phases that the subsequent regeneration step is intended to remove.

Binder removal therefore represents a compromise between improved particle liberation and preservation of cathode chemistry. More aggressive treatment can enhance separation but may also increase impurity formation or structural damage. The optimal conditions must consequently be selected together with the requirements of the downstream recycling route.

### 3.3. Separating Cathode from Graphite: Flotation

Once the electrode particles have been sufficiently liberated, separation of cathode active material from graphite becomes a major challenge. Froth flotation is particularly attractive because it exploits the natural difference in surface hydrophobicity between graphite and many cathode materials. However, this selectivity is effective only when particle surfaces are sufficiently free of binder and electrolyte-derived films.

**Figure 5:**
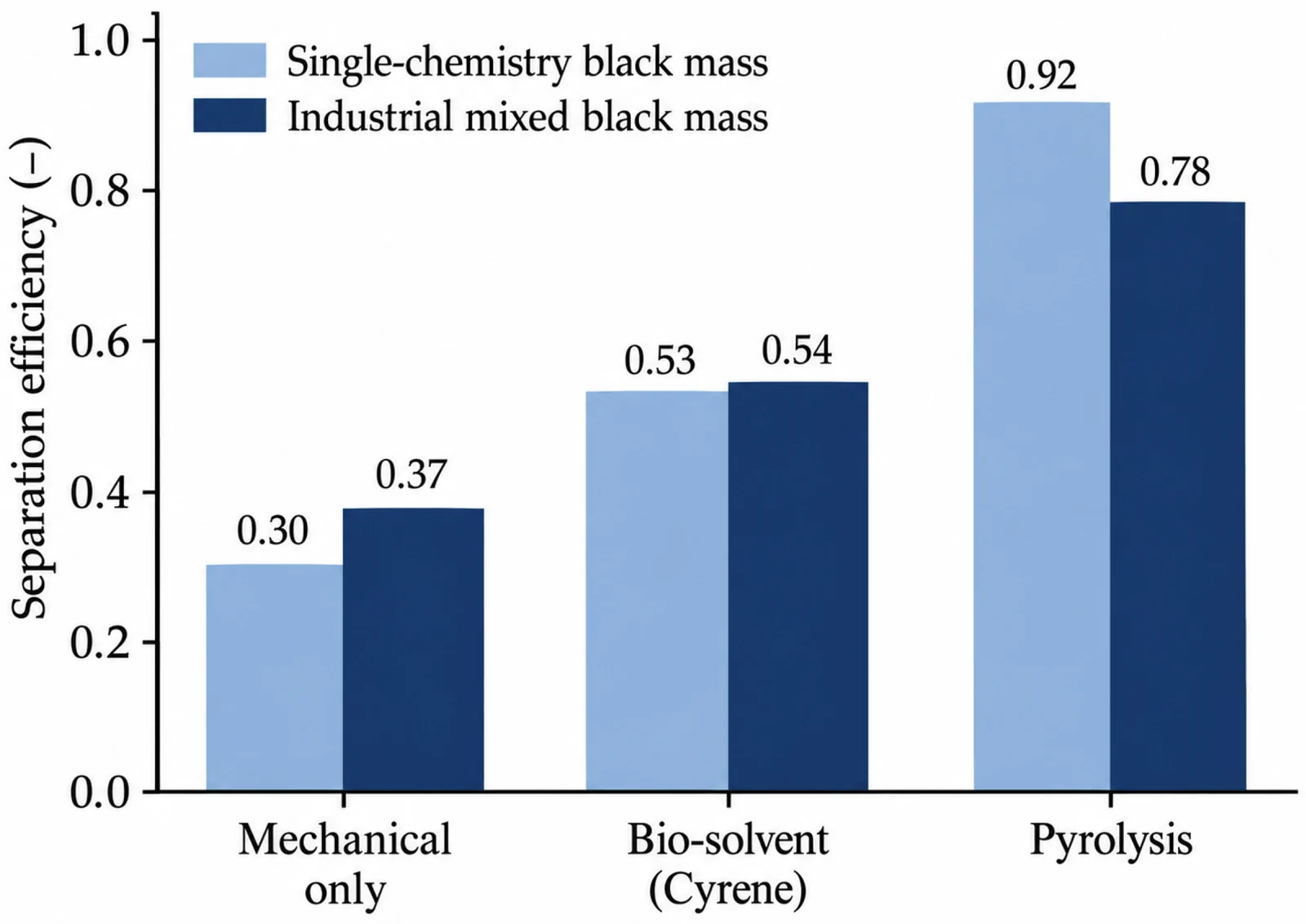
Effect of binder-removal pretreatment on the flotation separation efficiency of cathode active materials and graphite for single-chemistry and industrial mixed black mass. Figure created by the authors using data from [26].

This dependence is clearly illustrated by the separation efficiencies shown in Figure 5. With mechanical pretreatment alone, the separation efficiency was only 0.30 for single-chemistry black mass and 0.37 for industrial mixed black mass. Treatment with the bio-based solvent Cyrene™ improved the values to 0.53 and 0.54, while pyrolysis increased them further to 0.92 and 0.78, respectively [26]. These results confirm that surface conditioning before flotation is a decisive factor in determining separation performance.

Additional mechanical conditioning can further improve the result. Vanderbruggen et al. showed that attrition after pyrolysis enhances the separation of NMC particles from graphite [27]. In another study using industrial NMC523 black mass, roasting at 550 °C followed by a single rougher flotation stage produced a cathode fraction with 99.08% recovery and 95.92% grade [25]. Residual Al, Fe, and Cu present in the black mass captured fluorine as stable metal fluorides, and no HF was detected during roasting [25]. A related roasting–flotation route achieved a cathode grade of 91.6% at 92.6% recovery and a graphite grade of 84.6% at 82.7% recovery [28].

Comparable separation challenges are observed for LFP. For LFP black mass, sieving below 250 µm removed 71% of Cu and 81% of Al foil impurities, after which flotation was used to divide the material into graphite-rich and LFP-rich fractions for direct recycling [29]. This indicates that no single pretreatment step is sufficient on its own; effective purification requires a sequence of size classification, surface conditioning, and physical separation.

The required purity level ultimately depends on the intended downstream route. A cathode grade of approximately 90% may be acceptable for hydrometallurgical processing because residual impurities can be removed during dissolution and purification. For direct regeneration, however, the same grade may be insufficient because graphite, carbon black, binder residues, and Al or Cu fragments can remain in the regenerated product. Even a fraction described as nearly pure at approximately 96% grade still contains about 4% non-cathode material [25].

A second limitation is that the pretreatment conditions that most strongly improve separation can also introduce new chemical impurities. Pyrolysis enhances liberation and flotation efficiency, but it can convert fluorine-containing compounds into inorganic residues that remain with the solid material. Therefore, the central challenge in pretreatment is not simply to maximise recovery or separation efficiency. The objective is to produce a cathode-rich feed with sufficiently high chemical and phase purity while preserving the structural features required for subsequent regeneration. This balance between liberation, impurity control, and structural preservation ultimately determines whether black mass can be used only as a source of elements or as a feedstock for recovery of functional cathode material.

### 3.4. Mixed NMC/LFP Black Mass: Flotation Bottlenecks and Chemistry Separation

Industrial black mass increasingly contains NMC and LFP together, whereas most flotation studies, including most of those in Table 2, were performed on single-chemistry feeds. Mixed feeds introduce bottlenecks that do not arise in single-chemistry systems. First, froth flotation separates graphite from cathode particles on the basis of hydrophobicity, but it does not by itself separate one cathode chemistry from another, and LFP behaves differently from layered oxides. In a systematic comparison under identical reagent conditions, about 85% of LFP responded to the froth in single-material tests, compared with less than 8% of LCO and LiMn_2_O_4_; in a mixed cathode–graphite system, the froth recovery of LFP (82%) even exceeded that of graphite (40%), whereas only small amounts of the layered oxides floated. This behaviour was attributed to the combination of the ultrafine particle size of LFP, which favours entrainment, and its surface properties [30]. In mixed NMC/LFP black mass, LFP can therefore contaminate the graphite concentrate while NMC reports to the tailings, so that a single rougher stage produces partially mixed streams rather than a clean cathode and a clean graphite product.

Second, the thermal pretreatment windows of the two chemistries differ. Oxidative roasting of NMC523 black mass preserves the layered structure only up to about 550 °C, with rock-salt NiO appearing at 600 °C [25], whereas oxidative roasting of LFP black mass at 650 °C converts LiFePO_4_ into Li_3_Fe_2_(PO_4_)_3_ and Fe_2_O_3_ and removes the carbon [31]. A single thermal pretreatment of mixed feed is therefore unlikely to be optimal for the direct regeneration of both fractions. Third, the fine primary particles of LFP favour entrainment and heterocoagulation, which reduce selectivity even in LFP–graphite systems [29,30]; heterocoagulation between two cathode chemistries has prevented their flotation separation unless a dispersant was added, as shown for LiCoO_2_/LiMn_2_O_4_ mixtures [32]. Physical separation of NMC from LFP based on their different densities (about 4.7 and 3.6 g cm^−3^) or magnetic properties is conceivable, but, to the authors’ knowledge, no study has yet demonstrated an NMC/LFP separation that yields both fractions at a purity suitable for direct regeneration.

In current practice, mixed NMC/LFP black mass is therefore treated hydrometallurgically, where the presence of LFP can even be beneficial. Fe^2+^ released from LFP acts as a reductant for the layered oxide: with LFP replacing H_2_O_2_, complete Co and Li extraction with 88% Ni and 91% Mn was achieved from industrial NMC-dominated black mass [8]. Alternatively, oxalic acid can selectively dissolve 96% of the Li and 94% of the P from a mixed LFP–NMC feed while extracting only 13% of the Fe and 8% of the Mn, with no detectable Ni or Co [33]. These chemical routes, however, sacrifice the functional value of the LFP fraction. The technical gap for direct recycling is therefore twofold: a particle-level separation of NMC from LFP with a demonstrated purity specification, or, more realistically, identification and segregation of the chemistries before shredding.

### 3.5. Fluorine Impurities: Sources, Speciation and Mitigation

Fluorine deserves separate attention because, unlike graphite or metallic fragments, it is generated or redistributed by the pretreatment itself and can end up inside the regenerated cathode lattice. Its two main sources are the PVDF binder, which contains about 59 wt% F, and the LiPF_6_-based electrolyte, together with LiF and fluorophosphate species already present in the interphases of aged cells. On contact with moisture during storage, shredding and drying, LiPF_6_ hydrolyses to HF and fluorophosphate species, and during thermal binder removal PVDF decomposes with the release of HF, which reacts in situ with the black mass. The resulting fluorine is distributed among (i) gaseous HF and fluorinated organic species that leave with the off-gas, (ii) LiF and fluorophosphates, and (iii) poorly soluble metal fluorides such as AlF_3_ formed by reaction with Al foil fragments; AlF_3_, LiF and Li_x_PF_y_O_4_ have all been identified in thermally pretreated NMC622 powder [23]. The speciation determines the downstream risk. Gaseous HF is mainly a safety and emission problem, but fluorides that remain in contact with the cathode particles during relithiation and annealing can react with the oxide: in the direct regeneration of NMC from black mass, Al and F were incorporated into the cathode structure, with F substituting for lattice oxygen [23], a change that a subsequent anneal does not reverse (Table 1).

Several strategies limit the migration of fluorine toward the active phase. (1) Removing the electrolyte before any thermal step, for example by solvent or subcritical-CO_2_ extraction, recovers the fluorinated salt rather than converting it into HF; Rothermel et al. recovered 90% of the electrolyte in this way [34]. (2) Dissolving the binder in NMP or another solvent instead of decomposing it keeps fluorine in a separable polymer phase [14,16]. (3) When thermal treatment is unavoidable, conditions can be chosen so that fluorine is fixed in a defined phase rather than left as LiF on the cathode surface: during roasting of industrial NMC523 black mass at 550 °C, residual Al, Fe and Cu captured fluorine as stable metal fluorides and no HF was detected [25]. (4) Purification in non-aqueous media can remove most fluorine-containing species before relithiation, as shown for Ni-rich NMC black mass treated by the alkoxythermal process [35]. (5) Fluorine-free binders and design for recycling remove the source altogether [36]. The impurity risks associated with each pretreatment method are compared in the new “Impurity Risk” column of Table 2.

**Table 2:** Pretreatment methods and their fitness for subsequent recycling routes.

Method	Conditions	Outcome	Limitation	Impurity Risk	Suited to	Ref.
Reductive calcination (industrial)	Autothermic, O_2_-free	Accepts energised cells of mixed format	Destroys cathode structure	F partly volatilised (off-gas scrubbing); Al, Cu and P carried into the leach feed	Hydrometallurgy	[24]
NMP dissolution + sonication	Laboratory scale	Clean LFP powder from foil	Solvent cost and toxicity	Low F transformation (binder dissolved); residual NMP; Al fragments if foil is damaged	Direct regeneration (laboratory)	[14,16]
Calcination	500 °C, 3 h	PVDF and carbon removed	F retained as inorganic fluoride residues (Section 3.5)	High: AlF_3_, LiF and Li_x_PF_y_O_4_ remain on particles [23]	Direct regeneration; hydrometallurgy	[21,23]
Mechanical pretreatment + flotation	No binder removal	Separation efficiency 0.30–0.37	Binder blocks selectivity	Binder, electrolyte residues and graphite remain on particles	Hydrometallurgy	[26]
Pyrolysis + flotation	—	Separation efficiency 0.78–0.92	Emissions; F species	Medium–high: inorganic F residues and pyrolytic carbon	Hydrometallurgy; direct route after purification	[26]
Roasting + flotation	Kerosene 200 g t^−1^, MIBC 150 g t^−1^	Cathode grade 91.6%, recovery 92.6%	Grade too low for direct route	Medium: F residues; collector oil on surfaces; ≈8% non-cathode material	Hydrometallurgy	[28]
Sieving + flotation (LFP)	<250 µm	71% Cu and 81% Al removed	Graphite–LFP split still partial	Medium: residual Cu and Al (29% and 19% of foil impurities), binder; LFP–graphite cross-contamination	Direct regeneration of LFP	[29]
Roasting + flotation (industrial NMC523 black mass)	550 °C, 2 h, air; n-dodecane + MIBC	Cathode recovery 99.08%, grade 95.92%; F fixed as metal fluorides	≈4% non-cathode residue	Low–medium: F fixed as metal fluorides, no HF detected [25]; ≈4% non-cathode residue	Direct regeneration	[25]

## 4. Metal-Centred Recovery: From Extraction Efficiency to Battery-Grade Precursors

Metal-centred recycling routes are designed primarily to recover the elemental constituents of spent cathodes rather than to preserve the original cathode structure. In this approach, the active material is intentionally decomposed into simpler chemical forms, after which valuable metals are separated, purified, and converted into reusable intermediates or battery-grade precursors. This strategy is well established for Co- and Ni-containing cathodes because the economic value of the recovered metals can justify the additional thermal and chemical processing required.

Pyrometallurgy and hydrometallurgy represent the two principal metal-centred approaches. Pyrometallurgy relies on high-temperature reduction and phase separation, whereas hydrometallurgy dissolves the active material and recovers metals through solution-based separation and precipitation. Both routes can achieve high elemental recovery, but they differ strongly in energy demand, reagent use, product form, impurity tolerance, and chemistry dependence.

### 4.1. Pyrometallurgy

Pyrometallurgical recycling is one of the most robust approaches for processing heterogeneous battery feed because it can tolerate variation in cell format, cathode chemistry, state of charge, and degree of mechanical contamination. Rather than preserving individual electrode materials, the process converts the battery components into a limited number of high-temperature phases from which valuable metals can subsequently be recovered.

In the Umicore-type shaft furnace, cells pass through a pre-heating zone below 300 °C that releases electrolyte, a plastics-pyrolysis zone at about 700 °C, and a final smelting-reduction zone. During this sequence, organic components and graphite contribute to the thermal and reducing environment of the furnace. Cu, Co, Ni and part of the Fe report to a metallic alloy, whereas Li, Al, Si, Ca and part of the Fe are transferred to the slag phase [4]. The resulting alloy therefore concentrates the transition metals of highest conventional market value, but Li is not directly recovered in the same product stream.

This distribution of elements is one of the main limitations of conventional smelting. Although Co, Ni, and Cu are efficiently concentrated, Li typically requires additional processing from the slag, while Al and other elements are generally not recovered as high-value battery materials. Georgi-Maschler et al. showed that an electric-arc variant combined with physical pretreatment can improve Li recovery by concentrating it for conversion into Li_2_CO_3_ [37]. Nevertheless, the defining feature of the route remains the destruction of the original cathode structure and the conversion of the battery into metallurgical intermediates.

Graphite and electrolyte also play a different role in pyrometallurgy than in direct recycling. Instead of being recovered as functional battery materials, they can act as reductants or fuels during thermal treatment [4,38]. This can reduce the need for external reducing agents, but it also means that potentially recoverable carbon material is consumed in the process. From a circularity perspective, the route therefore recovers selected elements while sacrificing much of the functional value originally contained in the electrodes.

The economics of smelting are strongly dependent on cathode chemistry. For Co-rich materials such as LCO and some early NMC compositions, recovery of Co and Ni can compensate for the high energy demand of the process. As Co content decreases, however, the value of the recovered alloy also declines. For LFP, where Fe and P have relatively low commodity value and no Co or Ni is present, conventional smelting provides limited economic incentive based on recovered metals alone. This makes LFP particularly poorly matched to a recycling strategy centred on alloy recovery.

Environmental performance is also chemistry- and process-dependent. Ciez and Whitacre found that pyrometallurgical processing of cells does not significantly reduce life-cycle greenhouse-gas emissions [6]. The high-temperature nature of the process, combined with the loss of Li and other components to slag or secondary streams, limits its environmental advantage when compared with routes that retain more of the original cathode value.

Industrial practice, however, is not limited to conventional smelting. Reductive calcination represents an important alternative. In this process, carbothermal reduction is promoted under controlled conditions without complete combustion of graphite, and substantial Li loss can be avoided. The product is not intended as the final recovered material, but rather as a conditioned feed for subsequent hydrometallurgical refining [24]. In this configuration, thermal processing serves as a pretreatment step that simplifies downstream separation rather than as a stand-alone recycling route.

This distinction is important because it shows that pyrometallurgy should not be considered as a single fixed process. Its role can range from full smelting and alloy production to selective thermal conditioning before hydrometallurgical recovery. The suitability of each configuration depends on the value distribution of the cathode chemistry, the heterogeneity of the feed, and the intended final product.

### 4.2. Hydrometallurgy: Leaching Efficiency Versus Product Quality

Hydrometallurgical recycling decomposes cathode materials through controlled chemical dissolution and transfers the constituent metals into solution, where they can be separated and recovered by precipitation, solvent extraction, crystallisation, or related operations. Compared with pyrometallurgy, this route generally provides greater flexibility in recovering Li and transition metals separately and can produce intermediates that are closer to the chemical forms required for cathode manufacturing.

However, the apparent simplicity of high leaching efficiency can be misleading. Dissolving more than 95% of a metal from the solid phase is only the first step in a much longer process. The dissolved metals must still be separated from impurities, adjusted to the required stoichiometry, converted into sufficiently pure compounds, and ultimately transformed into a precursor or active material that meets battery-manufacturing specifications.

Table 3 compiles representative leaching conditions reported in primary studies. Under appropriate conditions, inorganic and organic acids alike dissolve more than 95% of Li, Co, Ni and Mn. Examples include 4 M HCl at 80 °C for LiCoO_2_/mixed cathodes [39], 2 M H_2_SO_4_ with 10% H_2_O_2_ at 70 °C [40], and L-tartaric acid with 4% H_2_O_2_ at 70 °C for mixed NMC [41]. These results demonstrate that complete or near-complete dissolution of transition metals is not, by itself, the main technical limitation of hydrometallurgy.

The reductant is particularly important for layered oxides because Co and Mn may be present in oxidation states that are less readily soluble under acidic conditions. Without an appropriate reducing agent, dissolution becomes strongly element-dependent. For example, 1 M H_2_SO_4_ at 95 °C for 4 h dissolved about 93% of Li and 96.3% of Ni, but only 66.2% of Co and 50.2% of Mn [42]. The Li value for this experiment is reported as 93.4% in [18] and 93.1% in [4], illustrating the variability that can arise when primary data are reproduced in later reviews.

Organic acids are often presented as environmentally preferable alternatives to mineral acids because of their lower corrosivity and potentially lower toxicity. However, the designation “green” depends on the full process rather than on the identity of the lixiviant alone. Oxalic acid, for example, can precipitate Co directly as cobalt oxalate during leaching and may also evolve CO_2_ [18,43]. The statement that organic acids are inherently gas-free or environmentally benign is therefore too general and must be evaluated on a case-by-case basis.

Discrepancies between secondary compilations call for further caution. For DL-malic acid (1.5 M, 2% H_2_O_2_, 90 °C, 40 min, 20 g L^−1^), one review reports 93% Co and 94% Li in the main text but 90% Co and 100% Li in its table for the same experiment [18,44]. Likewise, the 4 M HCl leaching of mixed LiCoO_2_/LiMn_2_O_4_/NMC111 by Wang et al. [39] is reported in one review with 20 g L^−1^ solids and 97–98% recoveries [18], whereas another source gives 5% *w/v* solids and recoveries above 99% for all metals [4]. These inconsistencies show why quantitative comparisons should be traced back to the original primary studies rather than reproduced uncritically from review tables.

Another limitation is that leaching efficiencies are rarely normalised to reagent consumption. Most laboratory studies operate at relatively low solid-to-liquid ratios of approximately 5–50 g L^−1^, as summarised in Table 3. Under such dilute conditions, very high recovery values may be obtained at the expense of excessive acid and reductant consumption per kilogram of cathode.

For LiCoO_2_ leached in HCl according to Equation (1) [4],

2LiCoO_2_ + 8HCl → 2CoCl_2_ + Cl_2_ + 2LiCl + 4H_2_O (1)

the stoichiometric acid demand is 4 mol HCl per mol LiCoO2, corresponding to approximately 41 mol HCl per kilogram of cathode. A 4 M HCl solution used at a solid loading of 20 g L^−1^ supplies approximately 200 mol HCl per kilogram of cathode, corresponding to roughly a five-fold stoichiometric excess. This calculation illustrates that apparently excellent dissolution efficiencies can conceal poor reagent utilisation.

Higher pulp densities have been demonstrated. However, the primary data show that the reductant dose then becomes limiting: Jha et al. leached LiCoO_2_ from spent mobile-phone cells at 100 g L^−1^ in 2 M H_2_SO_4_ with 5 vol% H_2_O_2_ at 75 °C and recovered 99.1% of the Li but only 70.0% of the Co within 60 min [45]. Such conditions are more relevant to industrial operation because they reduce the amount of liquid that must be heated, pumped, neutralised, and treated as effluent. Nevertheless, the broader literature still tends to emphasise maximum extraction percentage rather than reagent efficiency, water consumption, or product yield per unit of process input.

This distinction becomes especially important when hydrometallurgy is evaluated as a route to closed-loop battery recycling. A process that dissolves 99% of Ni, Co, Mn, and Li but requires large excesses of acid, generates high volumes of wastewater, or produces impure mixed salts may be less effective in practice than a process with slightly lower extraction but higher selectivity and more direct conversion into battery-grade precursor.

For this reason, the critical question in hydrometallurgical recycling is not simply how much metal enters solution, but how much of that metal is ultimately recovered in a form that can re-enter cathode manufacturing. The next stage of the process therefore shifts from leaching efficiency to impurity control, selective recovery, precursor composition, and battery-grade product quality.

**Table 3:** Representative leaching systems for cathode materials. Values are taken from the original studies unless indicated otherwise.

Cathode	Lixiviant; Reductant	T; t	S/L	Li	Ni	Co	Mn	Product/Note	Ref.
LiCoO_2_ + LMO + NMC111	4 M HCl; none	80 °C; 1 h	20 g L^−1^ [a] or 5% *w*/*v* [b]	97% [a]; >99% [b]	97% [a]; >99% [b]	97% [a]; >99% [b]	98% [a]; >99% [b]	Cl_2_ evolution; conflicting secondary reports	[39]
Mixed cathode (Co 35.8, Ni 10.1 wt%)	1 M H_2_SO_4_; none	95 °C; 4 h	50 g L^−1^	93.4% [a]; 93.1% [b]	96.3%	66.2%	50.2%	Shows need for reductant	[42]
NMC-type (Ni 14.4, Mn 26.2 wt%)	2 M H_2_SO_4_; 10% H_2_O_2_	70 °C	33.3 g L^−1^	99.8%	98.6%	98.5%	98.6%	Sulphate leachate	[40]
Mixed NMC	L-tartaric acid; 4% H_2_O_2_	70 °C; 30 min	17 g L^−1^	99.07%	99.31%	98.64%	99.3%	Organic leachate; harder separation	[41]
NMC111	1.5 M citric acid; D-glucose	80 °C; 2 h	20 g L^−1^	99%	91%	92%	94%	Atom-economic route	[46]
LiCoO_2_	1.25 M citric acid; 1% H_2_O_2_	90 °C; 30 min	20 g L^−1^	100%	—	90%	—	Li and Co citrates	[47]
LiCoO_2_	1 M oxalic acid; none	95 °C; 150 min	15 g L^−1^	98%	—	97%	—	CoC_2_O_4_ precipitates in situ; CO_2_ evolved	[43]
NCA	4 M HCl; none	90 °C; 18 h	5% *w*/*v*	100%	100%	100%	—	Long leaching time	[48]

[a] Values as reported in [18]; [b] values as reported in [4].

### 4.3. From Leachate to Battery-Grade Product

The ultimate objective of hydrometallurgical recycling is not simply to transfer metals from the solid cathode into solution, but to return them to the battery value chain in a form that satisfies cathode-manufacturing requirements. From this perspective, the most relevant quantity is not the leaching efficiency alone, but the fraction of recovered material that can be converted into a precursor or active material of sufficient chemical purity, stoichiometric control, and electrochemical performance.

Leachates obtained from spent lithium-ion batteries are chemically complex and can contain not only Li, Ni, Co, and Mn, but also Al, Cu, Fe, F, P, and C originating from current collectors, binders, electrolyte decomposition products, conductive additives, and residual electrode materials. These impurities can influence precursor formation, crystal structure, cation ordering, surface chemistry, and ultimately electrochemical performance. Their effects are strongly concentration-dependent and specific to cathode chemistry.

This is illustrated by impurity studies on NMC622. An Al content of 0.2 at% produced the best rate capability and a capacity of 138.5 mAh g^−1^ after 100 cycles at 2 C, whereas increasing the Al concentration to 5 at% promoted formation of a Li/Mn superlattice and led to poorer electrochemical performance [49]. A similar non-linear effect was observed for carbon contamination. At 0.2 at%, carbon improved cycling performance, yielding 159.9 mAh g^−1^ with 97.9% capacity retention after 100 cycles at 0.33 C, although high-rate performance was reduced. At 5 at%, however, carbon caused severe cation disorder [50]. These results, reviewed in [51], demonstrate that the concept of “high purity” is not sufficiently precise for recycled cathode materials. Instead, impurity tolerance windows need to be defined for each cathode composition and for each relevant contaminant.

Once impurity concentrations are controlled, hydrometallurgical recovery can produce materials with electrochemical properties comparable to, and in some cases better than, conventional commercial cathodes. NMC111 resynthesised from recycled leach liquor delivered discharge capacities of 150, 145, 130, and 100 mAh g^−1^ at 0.5, 1, 2, and 5 C, respectively, while retaining 94% of its capacity after 100 cycles at 0.5 C [52]. In parallel, Li_2_CO_3_ with a purity of 99.9% has been recovered from NMC111 scrap [53], demonstrating that sufficiently controlled hydrometallurgical refining can produce high-purity lithium intermediates suitable for reuse in battery-material synthesis.

A particularly important distinction is the level at which the recovered material is validated. Many recycling studies stop at chemical analysis, phase identification, or coin-cell testing. However, these measurements do not necessarily demonstrate that the recovered product can perform under conditions relevant to commercial manufacturing. More convincing evidence is provided by studies in which recycled materials are evaluated in larger-format cells and directly benchmarked against commercial references.

One of the strongest examples is the work of Ma et al., where recycled NMC111 tested in 1 Ah cells in cooperation with a cell manufacturer reached 4200 cycles before declining to 80% capacity retention and 11,600 cycles before reaching 70%. These lifetimes were reported to be 33% and 53% higher, respectively, than those of the commercial control [54]. Such validation is particularly important because it moves the assessment beyond metal recovery and demonstrates that recycled precursors can be transformed into cathode materials that remain functional under extended cycling conditions.

**Figure 6:**
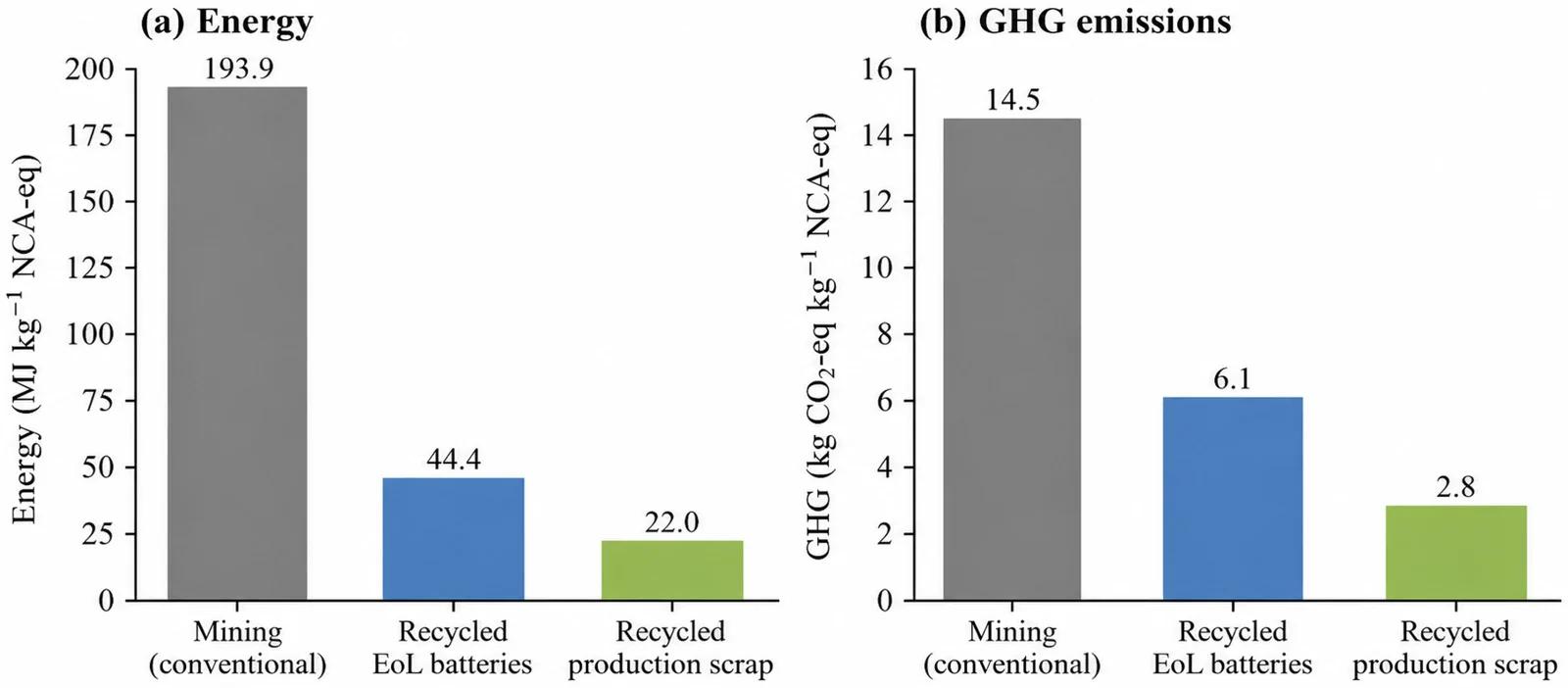
Gate-to-gate energy consumption (**a**) and greenhouse-gas emissions (**b**) for producing 1 kg of NCA-equivalent battery-grade material from mined resources and from recycled end-of-life batteries or production scrap at industrial scale (Redwood Materials, Nevada grid). Figure created by the authors using data from Machala et al. [24].

The industrial relevance of hydrometallurgical recovery is further illustrated by the energy and greenhouse-gas comparison shown in Figure 6. The figure compares production of 1 kg of NCA-equivalent battery-grade material from conventional mined resources with production from recycled end-of-life batteries and production from scrap. For mining-based refining, the reported gate-to-gate energy demand is 193.9 MJ kg^−1^ NCA-eq, whereas recycling end-of-life batteries reduces this requirement to 44.4 MJ kg^−1^ NCA-eq [24]. Greenhouse-gas emissions decrease in parallel from 14.5 to 6.1 kg CO_2_-eq kg−1 NCA-eq [24].

These reductions are important because they demonstrate that the benefit of hydrometallurgical recycling is not limited to recovering critical metals. When recycled feed is converted directly into battery-grade material, a substantial fraction of the upstream energy and emissions associated with primary extraction, beneficiation, and refining can be avoided. Figure 6 therefore illustrates the broader value of closed-loop material recovery: the recycled product substitutes not merely for an equivalent quantity of metal, but for part of the processing chain required to manufacture new cathode material.

The production-scrap pathway shown in Figure 6 also highlights the importance of feed quality. Production scrap is generally cleaner, more compositionally consistent, and less degraded than mixed end-of-life material, so less processing is required to return it to battery-grade form. This helps explain why manufacturing scrap can offer an especially favourable route for circular recovery, whereas end-of-life feed requires additional sorting, impurity removal, and compositional adjustment before equivalent product quality can be achieved.

The form in which metals are recovered also affects process efficiency. Refining the recovered transition metals into mixed (Ni,Co)SO_4_ rather than separating them into individual salts reduced energy consumption by a further 72.3% [24]. This observation suggests that maintaining useful chemical combinations where possible can reduce the number of separation and purification steps required before precursor synthesis. In other words, the most efficient recycling pathway is not necessarily the one that maximises the number of individually purified products, but the one that delivers the required cathode precursor with the minimum necessary processing.

Hydrometallurgical recovery is not limited to layered oxides. For LFP, resynthesis from recovered material has produced LiFePO_4_/C with a discharge capacity of 159.3 mAh g^−1^ at 0.1 C and 95.4% capacity retention after 500 cycles at 5 C [55]. This demonstrates that high-performance LFP can also be produced through dissolution and resynthesis, although the economic rationale differs from that of Ni- and Co-containing cathodes because the elemental value of Fe and P is much lower.

Taken together, these studies show that the critical transition in hydrometallurgical recycling occurs after leaching. High extraction efficiency is only an intermediate result. The more meaningful measure is whether the dissolved metals can be converted into compositionally controlled, impurity-tolerant, battery-grade products and whether these products retain their performance when tested against commercial materials. Figure 6 reinforces this point by showing that, when this conversion is achieved at industrial scale, recycled feed can significantly reduce both energy demand and greenhouse-gas emissions relative to primary material production.

## 5. Direct Cathode Regeneration: From Relithiation to Functional Cathode Recovery

Direct cathode regeneration differs fundamentally from metal-centred recycling because the objective is to preserve the existing cathode particles and restore their electrochemical functionality rather than decompose the material into individual elements. The potential advantage is the retention of part of the crystal structure, particle morphology, and processing history already embedded in the spent cathode. However, the feasibility of this approach depends strongly on the type and severity of degradation, the purity of the recovered cathode fraction, and the ability of the treatment to restore both composition and structure without introducing additional defects.

Three product levels are distinguished: regeneration restores the spent material toward its original composition, structure and morphology; upcycling deliberately changes its composition or particle morphology to yield a different cathode product; and resynthesis recovers a precursor by wet-chemical processing and re-sinters it into a new cathode. These product levels require different forms of validation: restoration of composition alone is insufficient if the regenerated material has not also recovered the structural and electrochemical properties required for practical reuse. Comprehensive descriptions of individual regeneration techniques are available elsewhere [56]; the discussion below focuses on how the effectiveness and limitations of these approaches depend on cathode chemistry and degradation state.

### 5.1. Solid-State Relithiation

Solid-state relithiation is conceptually the simplest regeneration route. Spent cathode powder is mixed with a lithium source, commonly Li_2_CO_3_ or LiOH, and subsequently annealed to restore lithium stoichiometry and repair structural defects. The method requires relatively simple equipment and avoids the complete dissolution of the cathode material.

For LFP, Chen et al. showed that treatment at 650 °C could restore the discharge capacity and energy density of spent material to values approaching those of fresh LFP, particularly at high current densities [57]. Later studies, however, showed that lithium replenishment alone does not necessarily guarantee complete regeneration. Xu et al. observed residual Li_3_PO_4_ and Fe_2_P after solid-state treatment and attributed these phases to inhomogeneous lithium distribution within the powder [14]. They also noted that accurate lithium dosing becomes difficult when the feed contains cells with different states of health and therefore different degrees of lithium deficiency [14].

Electrochemical testing at low current density may further overestimate regeneration quality. Ji et al. reported capacities of 146 and 152 mAh g^−1^ at 0.1 C for LFP regenerated using Li_2_CO_3_ and LiOH, respectively. After 400 cycles at 5 C, however, the corresponding capacities fell to 73 and 77 mAh g^−1^, whereas material regenerated with a multifunctional carbon-forming lithium source retained approximately 110 mAh g^−1^ [13]. These results show that initial capacity recovery is not sufficient evidence of full structural and functional restoration. Long-term cycling and high-rate performance are more sensitive indicators of residual defects and incomplete repair.

Solid-state relithiation is therefore most attractive when lithium deficiency is reasonably uniform and the original particle framework remains largely intact. Its main limitation is the difficulty of distributing the lithium source uniformly through heterogeneous industrial feed without resorting to prolonged or high-temperature treatment.

### 5.2. Aqueous and Hydrothermal Relithiation

Solution-based relithiation improves lithium transport by allowing Li^+^ to reach particle surfaces more uniformly than in conventional solid-state mixing. This can reduce compositional gradients and make the process particularly attractive for cathodes whose dominant degradation mechanism is lithium loss rather than extensive structural destruction.

For layered NMC, Shi et al. combined hydrothermal relithiation with a short annealing step and reported recovery of near-ideal stoichiometry, low cation mixing, and high phase purity [58]. In LFP, conventional hydrothermal relithiation at approximately 180 °C requires an autoclave operating above 11 bar. Xu et al. demonstrated that the temperature and pressure could be reduced substantially by adding citric acid as a reductant to 0.2 M LiOH. Comparable kinetics were obtained at 80 °C, while complete compositional recovery was achieved after 10 h at 70 °C and after 17 h at 60 °C under ambient pressure [14].

The reducing environment is essential for LFP. In the absence of citric acid, Fe-containing species were oxidised to Fe_2_O_3_ and Fe_3_O_4_, showing that reduction of Fe^3+^ is coupled to effective regeneration [14]. An additional advantage of solution relithiation is its ability to accommodate heterogeneous states of health. A mixture of cells at 40%, 50%, and 85% state of health reached the required stoichiometry within the same treatment because lithium uptake was self-limiting, and the relithiation solution could subsequently be reused [14].

Water, however, is not chemically neutral toward cathode surfaces. Even in LFP, unannealed relithiated material retained only 93.7% of its capacity after 100 cycles, an effect attributed partly to Li^+^/H^+^ exchange; a subsequent annealing treatment at 600 °C for 2 h was required to stabilise the regenerated material [14]. The problem becomes more severe for layered oxides. Proton intercalation can promote cation rearrangement and formation of impurity phases, with the effect increasing as the Ni content rises [59]. Li^+^ leaching into water is also greater for NMC532 than for LFP and increases with increasing Ni concentration [15].

For highly Ni-rich cathodes, aqueous treatment may therefore produce additional surface degradation rather than simply repairing existing defects. Gupta et al. found that impurities further aggravated the degradation of Ni-rich NMC during high-temperature hydrothermal processing and proposed a non-aqueous alkoxythermal route operating at 80 °C, which also removed most fluorine-containing species [35].

These results suggest that solution relithiation is particularly compatible with LFP and remains technically viable for low- and intermediate-Ni NMC, whereas increasing Ni content makes proton-free or non-aqueous environments progressively more attractive.

### 5.3. Molten-Salt and Eutectic Relithiation

Eutectic Li salts provide a proton-free, Li-rich liquid at ambient pressure. Shi et al. relithiated NMC523 with ≈40% Li loss and ≈50% capacity loss in a LiOH–LiNO_3_ eutectic at 300 °C for 2–4 h and then annealed the powder with excess Li_2_CO_3_ at 850 °C in O_2_. Composition, layered structure, capacity, cycling stability and rate capability returned to the levels of pristine material harvested from the same commercial cells: after 4 h relithiation, the regenerated NMC523 delivered 149.3 mAh g^−1^ at 1 C and 134.6 mAh g^−1^ after 100 cycles, against 146.6 and 130.4 mAh g^−1^ for the pristine reference [22]. The shorter 2 h treatment gave inferior cycling, which the authors attributed to residual cation mixing in the particle bulk. Later work extended the salt systems to LiOH–Li_2_CO_3_ [60], ternary melts [61], compositions adapted to highly degraded cathodes [62], organic eutectics for extremely low state-of-health Ni-rich material [63] and ionothermal lithiation [64].

For NMC811, He et al. used a LiOH–LiNO_3_–lithium salicylate eutectic (450 °C, 3 h) and sintering at 750 °C with 5 mol% excess LiOH. The NiO phase was eliminated, Li/Ni mixing fell from 6.5% to 3.3%, and the capacity at 0.1 C rose from 105.7 to 196.0 mAh g^−1^. At 1 C the material retained 133.3 of 175.4 mAh g^−1^ (76.0%) after 200 cycles [21]. No pristine NMC811 was tested, and the authors describe the work as laboratory-scale. A related study regenerated highly degraded NMC523 in a LiCl–LiNO_3_ eutectic with CoO and MnO_2_ additives, starting with a step at 300 °C for 4 h; it reported 165 mAh g^−1^ and 84% retention after 300 cycles at 1 C and claimed performance superior to pristine NMC523 [65]. Where transition-metal oxides are added only to compensate for transition-metal loss and the original stoichiometry is restored, the process remains regeneration in the sense of Section 1.2; where the additions change the composition, it becomes upcycling. In general, solidified salt must be washed off after molten-salt treatment [22]; washing can extract Li^+^ and lattice oxygen and reduce surface Ni^3+^ to Ni^2+^, so a further tempering step is often added, which has motivated single-step solid-state alternatives [25].

Comparing the eutectic studies on NMC523 reveals a pattern that is easily overlooked. Whatever salt system is used for relithiation, ranging from 200 °C (LiI–LiOH with Co_2_O_3_/MnO_2_) through 300 °C (LiOH–LiNO_3_) and 400 °C (LiOH–LiNO_3_–CH_3_COOLi) to 440 °C (LiOH–Li_2_CO_3_), the reported protocols are followed by annealing at 850 °C for 4–12 h, and the first-cycle capacities converge at about 146–150 mAh g^−1^ [22,60,61,62]. The eutectic therefore lowers the temperature of the relithiation step, but the thermal budget is dominated by the subsequent crystallisation anneal, which is comparable to that of conventional solid-state synthesis. The energy advantage of molten-salt regeneration over resynthesis lies mainly in avoiding dissolution, precipitation and precursor calcination, not in a low-temperature process overall. Process parameters are also inconsistently reported in secondary sources: the anneal for the LiI–LiOH route is given as 5 h in one review [56] and as 10 h in another publication [65], whereas the primary study describes a single, continuous heating programme [62].

### 5.4. Multifunctional Lithium Sources and Electrochemical Routes

An alternative strategy is to use lithium-containing reagents that perform several functions simultaneously. This is particularly relevant for LFP, where regeneration may require not only lithium replenishment but also reduction of Fe^3+^ and restoration of the conductive carbon coating.

Ji et al. used 5 wt% 3,4-dihydroxybenzonitrile dilithium (Li_2_DHBN) and treated the spent LFP at 800 °C for 6 h under Ar/H_2_. The reagent supplied lithium, its cyano-containing functionality assisted in removing Fe(III), and its pyrolysis regenerated a 4–5 nm carbon coating. FePO_4_ disappeared, the fraction of anti-site defects decreased from 2.4% to 1.2%, and the capacity at 0.1 C increased from 102 to 157 mAh g^−1^. The regenerated material retained 88% of its capacity after 400 cycles at 5 C and delivered 61 mAh g^−1^ at −20 °C [13].

The improved functionality comes at the cost of a substantial thermal requirement. Treatment at 800 °C for 6 h is considerably more energy intensive than aqueous relithiation followed by annealing at 600 °C for 2 h [14]. In addition, the lithium reagent itself is synthesised from LiH in tetrahydrofuran [13], so its upstream material and energy requirements need to be considered when assessing the overall environmental advantage.

Electrochemical approaches seek to avoid such thermal treatments altogether. Functionalised prelithiation separators, for example, can provide lithium electrochemically and have demonstrated the principle of electrode-level regeneration [66]. At present, however, these methods remain at an early stage and have not yet demonstrated the feed tolerance, scale, or process robustness required for treatment of industrial black mass.

### 5.5. Upcycling: When Regeneration Changes the Product

For severely degraded cathodes, restoring the original composition and morphology may be less practical than deliberately converting the spent material into a different cathode product. This distinction is especially important for Ni-rich NMC, where secondary-particle cracking, surface reconstruction, and compositional instability can make complete restoration difficult.

Spent polycrystalline NMC811 has been converted into single-crystal NMC622 through Co and Mn supplementation and subsequent sintering. The resulting material delivered 163.0 mAh g^−1^ and retained 93.9% of its capacity after 100 cycles at 1 C [67]. In another study, Ce-doped single-crystal NMC811 achieved 159.5 mAh g^−1^ at 5 C and 88.17% retention after 300 cycles at 1 C. Under the same test conditions, a commercial NMC811 reference delivered 146.3 mAh g^−1^ and 79.93% retention [68]. Ni-rich spent cathodes have also been converted into single-crystalline Li-rich oxides [69].

These examples demonstrate that spent cathode material can serve as a feedstock for high-performance products, but they should not be interpreted in the same way as restoration of the original material. Conversion from NMC811 to NMC622 changes the transition-metal ratio and reduces the Ni content, while doped or Li-rich products introduce compositions that may not correspond to the original commercial specification. Each product therefore requires independent qualification before reuse in battery manufacturing.

Upcycling also tends to be composition-specific. The amount of Co, Mn, Li, or dopant required depends on the initial feed chemistry and degradation state, making the approach difficult to apply directly to heterogeneous mixed black mass. Its strongest application may therefore be in well-characterised and segregated waste streams where the initial composition is known.

### 5.6. From Laboratory Powder to Industrial Feed

One of the largest gaps between direct regeneration research and industrial recycling lies in the nature of the feed material. Most laboratory studies begin with manually disassembled, single-chemistry cells from which the cathode powder is carefully harvested [14,21]. Industrial black mass is considerably more complex and can contain AlPO_4_, AlF_3_, Li_3_PO_4_, LiF, LixPF_y_O_4_, Li_2_CO_3_, residual graphite, conductive carbon, binder residues, and metallic contaminants. During regeneration, Al and F can become incorporated into the cathode structure, including substitution of F for lattice oxygen [23]. A comparison of the direct regeneration, upcycling, and resynthesis routes for LFP and NMC cathodes is presented in Table 4.

Only a limited number of studies have approached realistic processing conditions and scales. Ji et al. regenerated industrial NMC523 black mass using an all-dry plasma-assisted mechanochemical process that converted Al and Na impurities into functional dopants and produced a single-crystal material, i.e., an upcycled product in the sense of Section 1.2. The process was demonstrated on kilogram-scale batches, and the regenerated material showed 97.1% capacity retention over 1000 cycles in a 2 Ah pouch cell [70]. This represents an important step beyond conventional coin-cell demonstrations because both the feedstock and the electrochemical validation are closer to industrial conditions.

Li et al. used flotation-separated industrial NMC523 black mass with approximately 20% lithium deficiency and Li/Ni mixing above 5%. Solid-state relithiation with Li_2_CO_3_ at a Li:M ratio of 1.15 involved treatment at 750 °C for 3 h followed by 900 °C for 10 h. The Li/Ni mixing decreased to 1.63%, while the discharge capacity at 0.1 C increased from 116.4 to 139.3 mAh g^−1^, with 91.85% capacity retention after 100 cycles at 0.1 C [25]. The experiment nevertheless used coin cells with only 2–3 mg active-material loading and did not include a pristine material tested under identical conditions. 

These examples illustrate an important distinction between demonstrating that industrial black mass can be regenerated and demonstrating that the resulting material can substitute for a commercial cathode. The strength of the available evidence depends not only on electrochemical performance, but also on the quality of the benchmark. Some studies compare regenerated material with pristine material harvested from the same cell type [14,22], others use a commercial cathode tested under identical conditions [68], while several compare only with the spent material [21,25] or with an insufficiently defined pristine reference [65]. A commercial benchmark tested under the same protocol provides the most direct evidence that a regenerated cathode could replace a purchasable material.

The realism of electrochemical validation also varies considerably. Coin half-cells with only a few milligrams of active material and test durations of approximately 100–400 cycles remain common [13,14,21,25]. Such tests are useful for identifying intrinsic material recovery, but they do not reproduce the electrode loading, electrolyte limitation, pressure conditions, calendar ageing, and full-cell interactions relevant to practical battery operation. Automotive applications typically require much longer durability, including approximately 1000 cycles and long calendar lifetimes at the pack level [71]. Among the studies retained in this review, Xu et al., using electrodes with 19 mg cm^−2^ loading and graphite pouch cells [14], and Ji et al., using 2 Ah pouch cells over 1000 cycles [70], provide comparatively more realistic validation.

A further limitation is the incomplete accounting of process inputs. Excess lithium is frequently introduced during regeneration, including 4% excess Li_2_CO_3_ [14], 5 mol% excess LiOH [21], and a Li:M ratio of 1.15 [25]. Other approaches use organic lithium reagents synthesised under inert conditions [13] or transition-metal oxides to correct the composition of degraded cathodes [65]. These additions can materially affect both process cost and environmental burden, yet they are not always incorporated into mass or energy balances.

The thermal demand of direct regeneration also varies widely. Reported treatments range from solution relithiation at 60–80 °C followed by 2 h annealing at 600 °C [14], to 6 h at 800 °C for multifunctional solid-state regeneration [13], and to sequential treatments of 3 h at 750 °C and 10 h at 900 °C for industrial NMC523 black mass [25]. Direct regeneration should therefore not be considered inherently low-energy. Its energy advantage depends on the cathode chemistry, the extent of degradation, the purity of the feed, and the amount of structural repair required.

Taken together, current studies show that direct regeneration can restore high electrochemical performance, but the strength of this conclusion is highly dependent on feed realism, benchmark selection, cell format, cycle duration, process inputs, and thermal history. Future work should therefore combine realistic black-mass feedstocks with quantitative impurity analysis, representative electrode loadings, Ah-scale full-cell testing, direct comparison with commercial cathodes, and complete reporting of material and energy inputs. These criteria are necessary to determine whether a regeneration process is not only technically successful at laboratory scale, but also suitable for industrial cathode-to-cathode recycling. 

## 6. Chemistry-Resolved Assessment: NMC Versus LFP, the Anode, and Environmental–Economic Evidence

The preceding analysis shows that no single recycling route is optimal for all lithium-ion battery chemistries. The suitability of pyrometallurgy, hydrometallurgy, direct regeneration, or upcycling depends on the elemental value of the cathode, the dominant degradation mechanisms, feed purity, and the extent to which the original functional structure can be preserved. These differences are particularly clear when Ni-rich NMC and LFP are compared. The two chemistries occupy opposite ends of the recycling-value spectrum: Ni-rich NMC retains substantial transition-metal value but suffers complex structural degradation, whereas LFP has relatively low elemental value but often preserves an olivine framework that can be restored directly. The anode must also be considered because graphite separation affects cathode-feed purity and because recovery of the anode contributes materially to overall circularity.

### 6.1. Chemistry-Dependent Route Selection: Ni-Rich NMC Versus LFP

For Ni-rich NMC, metal-centred recovery remains strongly supported by both material value and industrial maturity. The cathode contains economically significant amounts of Ni and residual Co, and hydrometallurgical processing can recover these elements as battery-grade mixed sulfates with substantially lower environmental impact than primary mining and refining [24]. Resynthesised cathodes derived from recycled precursors have also been validated in Ah-scale cells [54], providing evidence that hydrometallurgical recovery can return material to the battery supply chain rather than merely extract metals.

Direct regeneration of Ni-rich NMC faces several chemistry-specific barriers. First, extensive secondary-particle cracking cannot be corrected by simple relithiation [19]. Second, increasing Ni content makes the layered oxide progressively more sensitive to proton exchange and aqueous treatment, limiting conventional hydrothermal approaches [59]. Third, industrial black mass introduces Al, F, graphite, binder-derived species, and other impurities that can become incorporated into the regenerated material [23]. These challenges become more severe as the cathode approaches NMC811 and higher-Ni compositions.

A further difficulty is that regeneration attempts to restore a product whose commercial specification may already have changed. Cathode development is moving toward altered particle morphologies, including single-crystal materials, modified coatings, and increasingly composition-specific designs. A cathode recovered from an older cell may therefore be chemically restored but still not correspond to the current commercial product specification. This helps explain why a significant fraction of recent Ni-rich recycling research has shifted toward upcycling rather than strict regeneration [67,68].

For heterogeneous, mixed, or heavily degraded Ni-rich feed, hydrometallurgical recovery to mixed precursors therefore offers a robust route because the original particle structure does not need to be preserved. Direct regeneration is more attractive for clean, sorted, single-chemistry material with moderate degradation, such as manufacturing scrap or well-characterised fleet-return batteries, where structural damage and contamination remain limited.

LFP presents a different recycling problem. The absence of Ni and Co substantially reduces the value obtainable through conventional metal-centred processing, while the olivine structure can remain largely intact even after considerable capacity loss. Under these conditions, preserving and restoring the functional cathode material can be more valuable than decomposing it into Fe-, P-, and Li-containing intermediates.

Available techno-economic studies support this distinction, although the absolute values are scenario-dependent. In EverBatt-based modelling, pyrometallurgical and hydrometallurgical processing of LFP were associated with costs of approximately 3.4 and 2.4 $ kg^−1^ cell, compared with estimated revenues of only 0.8 and 1.0 $ kg^−1^, respectively. Direct regeneration was estimated to cost approximately 2.1 $ kg^−1^ while generating 3.2 $ kg^−1^ in revenue, corresponding to a modelled margin of about 1.1 $ kg^−1^ [14]. Ji et al., using Chinese market prices and assuming a residual lithium content of x = 0.8 in LixFePO_4_, estimated profits of 945 $ t^−1^ for hydrometallurgy and 2556 $ t^−1^ for direct regeneration [13].

These values should not be interpreted as universally transferable profitability estimates. The disagreement between the studies concerning hydrometallurgical profitability arises partly from different price assumptions and from the residual Li content assigned to the spent LFP, to which hydrometallurgical revenue is sensitive [13,14]. Direct-regeneration economics also depend on whether the regenerated product can be qualified and sold at a value close to that of virgin LFP.

The more robust conclusion is therefore qualitative rather than absolute: for Ni-rich NMC, a substantial share of recycling value remains associated with recoverable transition metals, whereas for LFP the economic case increasingly depends on retaining the value of the functional cathode material itself. Cathode chemistry consequently changes not only the technical feasibility of regeneration but also the economic meaning of recycling.

### 6.2. What Happens to the Anode?

Although this review is cathode-centred, graphite must be included because its separation determines cathode-feed purity and its recovery materially affects the mass balance and environmental performance of a closed loop. Graphite makes up a large share of cell mass but is burned as a reductant in smelting [4], often left in leach residues, or downcycled. A flotation-derived carbon concentrate (92 wt% C at 89% recovery) has, for example, been used as a secondary carbon source for magnesia–carbon refractories [72]. Graphite is also absent from EU element-specific recovery targets [7]. Battery-grade regeneration is nevertheless well demonstrated at laboratory scale and has been reviewed comprehensively [73]. Rothermel et al. showed that graphite recovered after subcritical-CO2 electrolyte extraction outperformed a commercial synthetic graphite, while 90% of the electrolyte was recovered [34]. More recent purification routes reach 99.6–99.7 wt% purity and capacities of 340–360 mAh g^−1^ with high retention [74,75,76]. A combined thermal and wet-metallurgical route that leaches Li selectively in water before acid washing gave regenerated graphite with 350.2 mAh g^−1^, 90.5% initial Coulombic efficiency and better rate performance than commercial graphite [77].

Two critical points apply. First, several studies report capacities above the theoretical 372 mAh g^−1^ of LiC_6_. Examples are 397.03 mAh g^−1^ after 50 cycles at 0.1 C [75] and 423.8 mAh g^−1^ at 0.5 C for regenerated manufacturing-scrap graphite [78]. Such values indicate contributions from defects, surface functional groups or residual phases. They should prompt scrutiny of first-cycle efficiency and full-cell behaviour rather than be taken as evidence of superior quality. Second, spent graphite contains Li-bearing SEI and intercalated Li, so graphite purification can be integrated with Li recovery, either by selective water leaching [77] or within stepwise purification that yields metal by-products [74]. Silicon–graphite composite anodes, whose Si fraction undergoes large volume changes [15], add oxidised Si phases to the anode stream. To the authors’ knowledge, and within the literature search, no systematic recycling study of commercial Si–graphite anodes has been reported; this is flagged as a gap.

### 6.3. Environmental and Economic Evidence: Consistent in Direction and Inconsistent in Magnitude

Environmental and economic performance is a key factor in determining whether the technical advantages of a recycling route translate into a practical benefit. Table 5 summarises the main quantitative environmental and techno-economic results reported for pyrometallurgical, hydrometallurgical, and direct-regeneration routes for different cathode chemistries. Taken together, these studies show a generally consistent direction: direct regeneration can substantially reduce energy demand when the original cathode structure is retained, while industrial hydrometallurgical recycling of Ni-containing materials can reduce both energy consumption and greenhouse-gas emissions relative to primary production [24].

For LFP, direct regeneration has been estimated to require only a fraction of the energy associated with hydrometallurgical processing. One study reported energy requirements of 3.5 MJ kg^−1^ cell for direct regeneration and 30.6 MJ kg^−1^ cell for hydrometallurgy, meaning that the direct route required approximately 11% of the hydrometallurgical energy demand [14]. Another analysis reported 3.16 and 19.57 MJ kg^−1^ cell, respectively, corresponding to approximately 16% [13]. As shown in Table 5, the absolute values differ substantially even between studies using the same general EverBatt modelling framework, and the studies also disagree on whether hydrometallurgical recovery of LFP is economically profitable [13,14]. For the same LFP chemistry and the same routes, the two EverBatt-based estimates differ by factors of 1.56 for hydrometallurgy (30.6 vs. 19.57 MJ kg^−1^), 1.52 for pyrometallurgy (18.4 vs. 12.14 MJ kg^−1^) and 1.11 for direct regeneration (3.5 vs. 3.16 MJ kg^−1^) [13,14].

For Ni-containing feed, industrial hydrometallurgical recycling shows a clear environmental advantage over mining-based production [24]. However, the magnitude of this advantage depends strongly on how the system is defined. Machala et al. reported that gate-to-gate assessments of circular refining can differ by more than 30% because of methodological differences [24]. Some assessments express the recovered output as avoided primary metals or metals in solution, whereas others consider battery-grade cathode salts or precursors as the functional product [24]. These outputs are not equivalent because they represent different positions in the battery-material value chain.

System boundaries further complicate comparison. Xu et al., for example, assumed unsorted feed for pyrometallurgical and hydrometallurgical processing but sorted LFP for direct regeneration [14]. Under such assumptions, the sorting burden is not distributed equally between the routes. Electricity supply is another major variable, with the greenhouse-gas footprint of the same industrial refining process potentially differing by as much as a factor of five depending on the grid mix [24].

The industrial NMC523 results included in Table 5 provide another example. An EverBatt 2023 assessment reported energy requirements of 2.88, 39.12, and 42.97 MJ kg^−1^ for direct regeneration, pyrometallurgy, and hydrometallurgy, respectively [25]. The hydrometallurgical value is approximately twice that reported for LFP in [13,14], but this difference reflects not only process efficiency but also cathode chemistry, feed composition, functional unit, and the particular model assumptions employed.

One way to reduce the influence of these methodological differences is to normalise the results of each study to its own hydrometallurgical baseline. On this basis, direct regeneration requires approximately 11% [14], 16% [13], and 7% [25] of the corresponding hydrometallurgical energy demand, calculated from the reported values. Thus, although the absolute energy values vary considerably, the relative direction of the result remains consistent.

Table 5 therefore demonstrates both the potential environmental advantage of value-preserving recycling and the difficulty of assigning universal numerical rankings to the available routes. Cross-study comparisons should only be made when functional units, system boundaries, feed quality, sorting requirements, electricity mix, and recovered-product specifications are sufficiently compatible. Where these conditions are not met, the results should be interpreted as directional rather than directly pooled.

### 6.4. Recommendations for Harmonised Life-Cycle and Techno-Economic Assessment

The spread of values in Table 5 shows that the main obstacle to comparison is methodological rather than technological. Building on the general requirements of ISO 14040/14044 [79], the following minimum conventions are proposed for future life-cycle assessment (LCA) and techno-economic analysis (TEA) of cathode recycling routes.

(1) Functional unit. Two functional units should be reported in parallel: 1 kg of spent cells (or of black mass of stated composition) entering the plant, which describes the waste-treatment service, and 1 kg of cathode active material or precursor of stated composition and specification leaving the system, which describes the product. Results per kWh should be added only when the energy content of the feed is known. The level of the output (metal, salt, precursor, regenerated cathode or validated cathode; Figure 2) must be stated, because outputs at different levels are not substitutable.

(2) System boundary and credits. The boundary should be stated explicitly as gate-to-gate (recycling plant only) or cradle-to-gate (including collection, transport, discharge and dismantling). Credits for avoided primary production should be reported separately from process burdens and calculated for the same product level as the recycled output.

(3) Sorting. The energy, materials and losses of chemistry identification, sorting and NMC/LFP separation must be included for every route that requires them. When one route requires sorted single-chemistry feed (typically direct regeneration) and another accepts mixed feed (typically pyro- or hydrometallurgy), a comparison is valid only if the sorting burden is either allocated to the route that requires it or included for all routes; the choice should be stated and a sensitivity case with mixed feed reported.

(4) Feed definition. Three feed classes should be distinguished: production scrap; sorted, single-chemistry end-of-life material; and mixed black mass of stated composition, including its NMC:LFP ratio and its graphite, Al, Cu and F contents. Results obtained for one class should not be transferred to another.

(5) Electricity and data sources. The grid mix and its emission factor (kg CO_2_-eq kWh^−1^), the model and its version (e.g., EverBatt 2020 or 2023), the plant capacity and the price year of the TEA should be reported. Because the greenhouse-gas footprint of the same refining process can vary up to five-fold with the grid mix [24], results for at least two grid mixes are recommended.

(6) Process inputs and yield. Reagent consumption (acid, reductant, Li source including the Li excess), water use, wastewater and the thermal budget (temperature and time of each step) should be reported per functional unit, together with the product yield, so that the assessment can be linked to gates G3 and G4 of the framework. These conventions are included in the minimum reporting set in Table 7.

### 6.5. Chemistry-Resolved Decision Matrix for Recycling Route Selection

The environmental and economic evidence summarised in Table 5, together with the preceding analysis of degradation mechanisms, feed purity, material value, and regeneration feasibility, shows that route selection cannot be reduced to a single universal hierarchy. A process that is favourable for one cathode chemistry may be poorly suited to another because the recoverable value and the type of degradation are fundamentally different.

To integrate these factors into a practical route-selection framework, Table 6 presents a qualitative decision matrix for the principal cathode chemistries and feed conditions considered in this review. The matrix links cathode chemistry with elemental value, degradation state, feed purity, structural preservation potential, and the relative suitability of metal-centred recovery and direct regeneration.

Numerical scores are deliberately avoided in Table 6 because sufficiently harmonised data on energy demand, processing cost, impurity tolerance, product quality, scale, and electrochemical validation are not available for every chemistry–route combination. As demonstrated by the results compiled in Table 5, even studies examining similar technologies can differ substantially in absolute values because of different functional units and system boundaries. A numerical ranking could therefore imply a level of precision that is not supported by the current evidence.

For LFP, the decision matrix reflects the low commodity value of Fe and P, the absence of Ni and Co, and the comparatively high potential to preserve and restore the olivine structure. Where the feed is sufficiently pure and the particle framework remains intact, direct regeneration can retain considerably more functional value than decomposition into individual elemental products [13,14].

For Ni-rich NMC, the balance shifts toward metal-centred recovery as degradation becomes more severe. Ni and residual Co maintain substantial elemental value, while microcracking, surface reconstruction, proton sensitivity, and impurity incorporation make direct restoration increasingly difficult [19,23,59]. Hydrometallurgical recovery is therefore particularly suitable for heterogeneous or heavily degraded Ni-rich material, whereas direct regeneration remains more plausible for clean, sorted, moderately degraded streams such as manufacturing scrap.

Low- and intermediate-Ni NMC occupy an intermediate region of the matrix. Their layered structures can often be restored when degradation is dominated by lithium loss and limited cation disorder, but the advantage of regeneration decreases as mechanical damage and contamination increase. In these cases, the choice between regeneration and resynthesis should depend on the actual state of the recovered cathode rather than on nominal chemistry alone.

Mixed black mass represents the most challenging feed condition considered in Table 6. When NMC, LFP, graphite, Al, Cu, binder residues, and electrolyte-derived compounds are present together, the decisive operation may be the separation that occurs before either regeneration or metal recovery. Direct regeneration requires a sufficiently pure and compositionally identified cathode stream, whereas hydrometallurgical processing is more tolerant of heterogeneous feed.

Taken together, Table 5 and Table 6 serve complementary purposes. Table 5 summarises the quantitative environmental and economic evidence available in the literature, whereas Table 6 translates the combined technical, chemical, and economic evidence into a qualitative route-selection framework. The two tables therefore show why the optimal recycling strategy should be selected according to cathode chemistry, degradation state, and feed quality rather than by applying a single process to all lithium-ion battery waste streams.

## 7. Toward Cathode-to-Cathode Circularity: Framework, Research Gaps and Conclusions

The preceding analysis shows that high elemental recovery alone does not necessarily correspond to effective closed-loop recycling. A process may recover more than 95% of Li, Ni, Co, or Mn and still fail to return a battery-grade material to the manufacturing chain. For this reason, recycling performance should be evaluated not only at the extraction stage, but across the entire sequence linking spent batteries to qualified material in new cells.

### 7.1. Why High Recovery Does Not Demonstrate Closed-Loop Recycling

Extraction efficiency measures the fraction of an element transferred from the solid feed into a recoverable phase or solution. Although this is an important process metric, it does not indicate how much of the recovered material ultimately reaches battery-grade specification, how much survives subsequent purification and synthesis, or whether the final product performs adequately in a new cell.

This distinction is particularly important for chemistries such as LFP, where much of the value is associated with the preserved functional cathode rather than with high-value transition metals. Current regulatory targets are largely element-based and therefore reward the recovery of Co, Ni, Cu, and Li, while giving much less attention to the recovery of functional cathode materials or graphite [7]. A process can therefore satisfy elemental recovery targets while still losing a substantial fraction of the embodied processing value contained in the original battery material.

Electrochemical validation introduces a second problem. Laboratory studies frequently demonstrate capacity recovery in half-cells at low current densities, but this does not establish that the regenerated material can substitute for a commercial cathode in realistic applications. Full-cell testing, representative electrode loading, extended cycling, and comparison with a commercial or pristine reference are required before claims of closed-loop functionality can be considered robust.

These considerations suggest that recycling performance should be assessed as a sequence of linked material and quality-retention steps rather than as a single extraction percentage.

### 7.2. The Cathode-to-Cathode Circularity Framework

**Figure 7:**
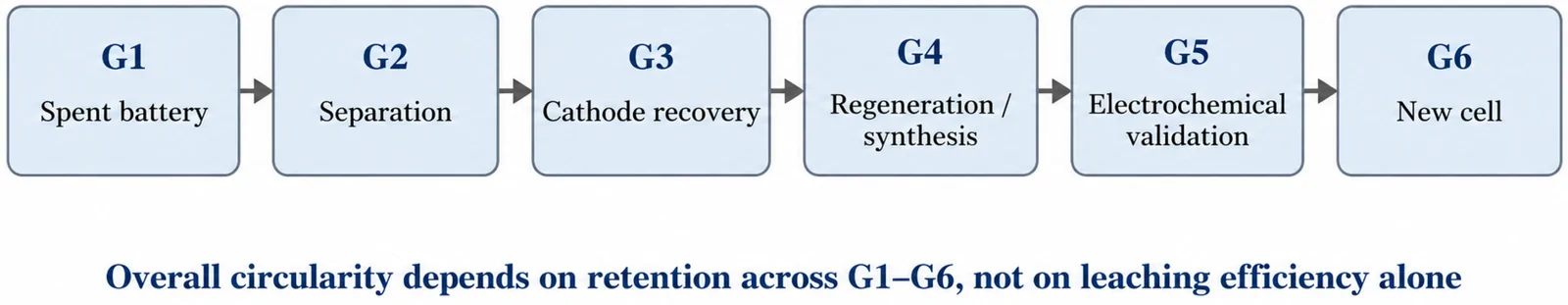
Proposed cathode-to-cathode circularity framework. Each gate (G1–G6) represents a measurable material- or quality-retention step, from chemistry identification and separation to electrochemical validation and reuse in new cells. Environmental and process inputs should be reported across the full chain.

Figure 7 presents the proposed cathode-to-cathode circularity framework. The framework evaluates recycling as a chain of six consecutive gates, each representing a measurable stage in the transition from spent battery to reused cathode material.

G1 concerns identification of the spent battery, including cathode chemistry and state of health. This information determines whether the feed is suitable for direct regeneration or requires more extensive processing.

G2 represents separation and purification. At this stage, the objective is to obtain a sufficiently pure, ideally single-chemistry cathode fraction with controlled levels of graphite, metallic contaminants, binder residues, and electrolyte-derived species.

G3 corresponds to cathode recovery and is expressed as the mass yield of cathode material or precursor recovered per unit of cathode input.

G4 evaluates whether regeneration or synthesis restores the required composition, phase structure, morphology, and impurity level relative to the intended material specification.

G5 concerns electrochemical validation. The recovered material should be tested in a representative full-cell configuration at realistic loading and benchmarked against a commercial or pristine reference.

G6 represents actual reuse, expressed as the fraction of recovered material that is successfully incorporated into newly manufactured cells.

The overall circularity of a recycling route is therefore determined by the combined performance across all six gates rather than by the extraction efficiency at a single stage. Losses at any point in the chain reduce the fraction of material that ultimately returns to battery manufacture. Energy demand, reagent consumption, excess lithium addition, wastewater generation, and greenhouse-gas emissions should also be reported across the same sequence. These quantities are only meaningfully comparable when the functional unit, system boundary, and electricity mix are clearly defined. Each item is assigned to the gate of Figure 7 to which it provides evidence, together with the quantity to be reported, its unit and the minimum specification that we consider acceptable for a claim of cathode-to-cathode recycling. Items 1–3 characterise the feed, items 4–7 the recovery and restoration steps, items 8–11 the electrochemical validation and items 12–13 reuse and the environmental and economic performance.

### 7.3. Minimum Reporting Set

Because inconsistent reporting is the main obstacle to comparing recycling studies (Section 4.2, Section 5.6 and Section 6.3), the requirements formulated in the preceding sections are consolidated here into an explicit minimum reporting set (Table 7). 

### 7.4. Applying the Framework: Two Illustrative Cases

Table 8 applies the six gates sequentially to two contrasting cases: the direct regeneration of LFP, represented by the most completely validated study among those reviewed [14], and the hydrometallurgical resynthesis of Ni-rich layered oxides, for which industrial data are available [24]. The evaluation proceeds gate by gate, and a gate is regarded as met only when the evidence is quantitative and was obtained on material representative of the following gate. The two routes fail at different gates. Direct regeneration of LFP is strong at G4 and G5, where composition, structure and long-term performance in graphite pouch cells were restored and benchmarked against fresh material, but its circularity is limited by G1–G3 and G6: the feed consisted of identified, manually dismantled single-chemistry cells, the purity of industrial LFP black mass was not addressed, and the mass yield and actual reuse were not reported. Hydrometallurgical resynthesis passes G1–G4 at industrial scale because it tolerates mixed feed and removes impurities in solution, but the Ah-scale validation available for resynthesised material concerns NMC111 [54], so G5 is only partly met for Ni-rich grades, and the fraction of recovered material that actually enters new cells (G6) is not reported. The framework therefore identifies, for each route, the specific evidence that is still missing, instead of ranking the routes by a single extraction percentage.

### 7.5. Research Gaps and Future Directions

Despite rapid progress in lithium-ion battery recycling, several barriers still prevent routine cathode-to-cathode circularity.

- **Chemistry identification and sorting. Mixed** NMC/LFP black mass remains a major challenge because direct regeneration generally requires a single-chemistry input. Automated identification at pack or cell level, supported by labelling and digital battery passports, could improve feed segregation before shredding [7,36]. Rapid identification technologies under investigation include handheld or in-line X-ray fluorescence and laser-induced breakdown spectroscopy of cells or black mass, and non-destructive electrochemical fingerprinting; incremental capacity analysis, for example, reliably distinguished LFP from layered-oxide chemistries in end-of-life cells, although identification became less reliable for heavily used cells with low SOH [80]. The battery passport required by Regulation (EU) 2023/1542 for EV, light-means-of-transport and industrial batteries above 2 kWh from February 2027 should make chemistry information available upstream of the recycler [7].
- **Purity specifications for direct-regeneration feed.** Tolerable concentrations of graphite, binder residues, Al, Cu, F, and other contaminants remain poorly defined for regenerated cathodes. Most systematic impurity studies have been performed for resynthesised materials rather than for direct regeneration [23,49,50,51].
- **Binder and electrolyte handling.** Pretreatment methods that improve particle liberation can simultaneously convert fluorine-containing components into persistent inorganic residues. Fluorine-free binders and design-for-recycling strategies may reduce this trade-off [26,36].
- **Regeneration of heterogeneous feed.** Solution relithiation can tolerate different states of health in LFP because lithium uptake can be self-limiting [14]. Equivalent strategies for mixed NMC grades or mixed NMC/LFP feed remain poorly developed.
- **Ni-rich cathode repair.** For high-Ni materials, relithiation alone cannot remove crack networks, severe surface reconstruction, or extensive interfacial damage. Approaches capable of repairing such defects without substantially changing the original composition remain limited [35,59].
- **Scale and independent validation.** Kilogram-scale regeneration and Ah-level full-cell testing remain uncommon [70]. Independent validation under industry-relevant protocols, comparable in rigor to the resynthesis validation reported in [54], is still needed for directly regenerated NMC and LFP.
- **Harmonised life-cycle and techno-economic analysis.** Reported environmental and economic results remain difficult to compare because of differences in functional units, sorting assumptions, system boundaries, grid mixes, and product definitions [13,14,24]. Conventions to address this are proposed in Section 6.4.
- **Future cathode and anode chemistries.** LMFP, Co-free high-Mn cathodes, and Si–graphite anodes have received, to the authors’ knowledge, comparatively little recycling attention. Their degradation and recovery behaviour should be characterised before large end-of-life volumes enter the recycling stream.
- **Batch consistency of recycled cathodes.** End-of-life feed arrives with a wide distribution of SOH and degradation modes, so recycled products, and directly regenerated products in particular, may vary more between production batches than virgin materials. Batch-to-batch variation in composition, cation mixing, impurity content and capacity is rarely reported; at least three independent batches should be characterised (Table 7), and SOH-based pre-grading or blending strategies for heterogeneous feed need to be developed.
- **Long-term and calendar ageing of recycled cathodes.** Validation of recycled materials is dominated by cycling tests of a few hundred cycles. Calendar ageing of regenerated or resynthesised cathodes, which may contain residual F, Al or surface defects that accelerate electrolyte oxidation and transition-metal dissolution during storage, has, to the authors’ knowledge, not been assessed systematically. Storage tests at elevated temperature and high state of charge, benchmarked against commercial material, are required before recycled cathodes can be qualified for automotive use [71].

### 7.6. Conclusions

The evolution of cathode chemistry changes the central question of lithium-ion battery recycling from simply asking which elements can be recovered to asking how much functional material can be returned to battery manufacture.

For LFP, degradation is dominated largely by lithium loss, Fe(III) formation, and anti-site defects while the olivine framework can remain substantially preserved. These defects can often be corrected by relithiation, reduction, and subsequent annealing. Available techno-economic studies therefore indicate a strong potential for direct regeneration of sorted LFP streams, although the result remains dependent on feed purity, product qualification, and the cost of separating LFP from other chemistries [13,14].

For Ni-rich NMC, the situation is more complex. Crack networks, surface reconstruction, proton sensitivity, and impurity uptake limit the effectiveness of simple direct restoration. Hydrometallurgical recovery to mixed precursors already has industrial validation and can reduce environmental impact relative to primary production [24,54]. For mixed or severely degraded Ni-rich feed, metal-centred recovery is therefore better supported by current evidence, while direct regeneration remains most appropriate for clean, well-characterised, moderately degraded streams.

Low- and intermediate-Ni NMC occupy an intermediate position. Both direct regeneration and resynthesis can be technically viable, and the choice should depend on the extent of structural damage, feed purity, and the quality of the recovered cathode fraction.

Across all chemistries, extraction efficiency alone is an incomplete measure of circularity. A meaningful closed-loop metric must connect feed identification, separation quality, material recovery, structural restoration, electrochemical validation, and actual reuse in new cells. The minimum reporting set in Table 7 is proposed as a practical first step toward such a metric.

## Figures and Tables

**Table 1:** Degradation modes of cathode materials and their reversibility by relithiation and short annealing. Assessments are based mainly on laboratory-cycled cells; for field-aged cells combining cycle and calendar ageing, see the discussion in Section 2.3.

Degradation Mode	Chemistries	Reversible by Relithiation + Anneal?	Evidence	Route Implication
Li inventory loss	All	Yes	NMC523: ≈40% Li loss restored [22]; LFP: 47.1% deficiency restored [14]	Direct regeneration
Fe(III)/FePO_4_ formation	LFP	Yes, with reductant	Citric acid or cyano-group reduction [13,14]	Direct regeneration
Fe/Li anti-site defects	LFP	Yes	4.81% → 1.5% [14]; 2.4% → 1.2% [13]	Direct regeneration
Li/Ni cation mixing	NMC, esp. high-Ni	Largely	6.5% → 3.3% in NMC811 [21]	Direct regeneration with higher thermal budget
Surface rock-salt layer	NMC	Yes, if thin	Reconverted to layered phase [21,22]	Direct regeneration
Carbon-coating loss	LFP	Not by Li supply alone	Requires a carbon source [13]	Multifunctional Li source
Microcracks, fragmentation	Ni-rich NMC	No (requires recrystallisation)	Crack propagation for x > 0.8 [19]; calendar-ageing cracks [20]	Upcycling or resynthesis
Impurity incorporation (Al, F)	NMC from black mass	No; F substitutes for O	[23]	Purification before regeneration

**Table 4:** Direct regeneration and upcycling of LFP and NMC cathodes compared with a resynthesis reference. Product level: R, regeneration; U, upcycling; S, resynthesis.

Cathode [Ref.]	Initial Degradation State	Method/Li Source	Temperature/Time	Structural Recovery	Capacity: Spent → Regenerated	Retention	Benchmark vs. Pristine	Scale
LFP [14] (R)	Up to 50% decay after 6500 cycles; 47.1% Li deficiency; 4.81% anti-site	0.2 M LiOH + 0.08 M citric acid; anneal with 4% excess Li_2_CO_3_	60–80 °C (5 h at 80 °C); 600 °C, 2 h, N_2_	Pure olivine; anti-site 1.5% (pristine 2.5%)	103 → 159 mAh g^−1^ (0.5 C)	<1% loss over 100 cycles; 150 mAh g^−1^ after 1000 cycles	Yes: 161 mAh g^−1^ (0.5 C), fresh cells	Lab; 19 mg cm^−2^; pouch cell
LFP [13] (R)	21.3 wt% FePO_4_; 2.4% anti-site; C-coating loss	Solid-state; Li_2_DHBN, 5 wt%	800 °C, 6 h, Ar/H_2_	FePO_4_ removed; anti-site 1.2%; 4–5 nm C	102 → 157 mAh g^−1^ (0.1 C)	88% after 400 cycles (5 C)	Composition vs. commercial LFP; electrochemical n.r.	Lab
NMC523 [22] (R)	≈40% Li loss; ≈50% capacity loss	LiOH–LiNO_3_ eutectic; anneal with excess Li_2_CO_3_	300 °C, 4 h; 850 °C, 4 h, O_2_	Layered structure; rock salt reconverted	149.3 mAh g^−1^ (1 C)	134.6 mAh g^−1^ after 100 cycles (90.2%)	Yes: 146.6 → 130.4 mAh g^−1^	Lab
NMC523 [60] [c] (R)	Spent	LiOH–Li_2_CO_3_ eutectic	440 °C, 4 h; 850 °C, 12 h	Layered	146.3 mAh g^−1^	89.06% after 200 cycles	n.r.	Lab
NMC523 [61] [c] (R)	Spent	LiOH–LiNO_3_–CH_3_COOLi	400 °C, 4 h; 850 °C, 5 h	Layered	150 mAh g^−1^	93.7% after 100 cycles	n.r.	Lab
NMC523 [62] [c] (R)	Highly degraded	LiI–LiOH + Co_2_O_3_, MnO_2_	200 °C, 4 h; 850 °C, 5 h [c] or 10 h [65]	Layered	≈150 mAh g^−1^	80% after 200; 73% after 300 cycles	n.r.	Lab
NMC523 [65] (R)	Highly degraded (after 300 cycles at 1 C)	LiCl–LiNO_3_ + CoO, MnO_2_	300 °C, 4 h; second step n.r.	Restored (DFT-supported)	165 mAh g^−1^	84% after 300 cycles (1 C)	Claimed superior	Lab
NMC811 [21] (R)	Li/TM 0.73; 5.15% NiO; 6.5% Li/Ni mixing; cracks	LiOH–LiNO_3_–Li salicylate + 5 mol% LiOH	450 °C, 3 h; 750 °C	NiO removed; mixing 3.3%	105.7 → 196.0 mAh g^−1^ (0.1 C)	76.0% after 200 cycles (1 C)	No	Lab
NMC523 black mass [70] (U)	Industrial; Al, Na, F impurities	All-dry plasma-assisted mechanochemistry + anneal	n.r.	Single-crystal layered	n.r.	82.6% after 300 cycles (4.5 V); 2 Ah pouch 97.1% after 1000 cycles	n.r.	kg batches; 2 Ah pouch
NMC523 black mass (flotation) [25] (R)	Industrial; ≈20% Li deficiency; Li/Ni > 5%; cracks	Solid-state; Li_2_CO_3_ (Li:M = 1.15)	750 °C, 3 h; 900 °C, 10 h, air	Li/Ni mixing 1.63%	116.4 → 139.3 mAh g^−1^ (0.1 C)	91.85% after 100 cycles (0.1 C)	No	Lab; 2–3 mg, coin cells
NMC811 → SC-NMC622 [67] (U)	Polycrystalline, multiphase	Sintering + Co/Mn	n.r.	Single crystal	163.0 mAh g^−1^	93.9% after 100 cycles (1 C)	Different product	Lab
NMC811 → Ce-doped SC-NMC811 [68] (U)	Spent polycrystalline	Eutectic + Ce doping	n.r.	Single crystal	159.5 mAh g^−1^ (5 C)	88.17% after 300 cycles (1 C)	Yes: commercial NMC811 146.3 mAh g^−1^ (5 C), 79.93%	Lab
NMC111 resynthesis (reference) [54] (S)	Mixed spent LIBs	Hydrometallurgical precursor + synthesis	n.r.	Void-rich particles	n.r.	1 Ah cells: 4200 cycles to 80%; 11,600 to 70%	Yes: +33%/+53% vs. commercial	Industrial-format test

[c] Process parameters as compiled in [56]; for the LiI–LiOH route, the anneal duration differs between secondary sources (5 h in [56]; 10 h in [65]), whereas the primary study describes a single, continuous heating programme [62]. n.r., not reported.

**Table 5:** Quantitative environmental and economic comparisons of recycling routes.

Study (Chemistry)	Functional Unit; Boundary	Energy	GHG Emissions	Economics	Key Caveat
Xu et al. 2020 (LFP) [14]	Per kg spent cell; EverBatt; 10,000 t yr^−1^ plant	Pyro 18.4; hydro 30.6; direct 3.5 MJ kg^−1^	Direct 0.7 kg CO_2_-eq kg^−1^ (26.6% of pyro, 27.7% of hydro)	Profit −2.6/−1.4/+1.1 $ kg^−1^	Direct route assumed to receive sorted feed
Ji et al. 2023 (LFP) [13]	Per kg spent cell; EverBatt 2020; Chinese prices	Pyro 12.14; hydro 19.57; direct 3.16 MJ kg^−1^	1.96/1.49/0.59 kg CO_2_-eq kg^−1^	Profit: hydro 945, direct 2556 $ t^−1^ (x = 0.8)	Sign of hydro profit opposite to [14]
Li et al. 2026 (NMC523 black mass) [25]	Per kg black mass; EverBatt 2023; 1000 t yr^−1^	Pyro 39.12; hydro 42.97; direct 2.88 MJ kg^−1^	Direct ≈ 1/2 of hydro, ≈1/3 of pyro	Direct 6.8× (vs. pyro) and 2.4× (vs. hydro) more profitable	No pristine benchmark for the product
Machala et al. 2025 (mixed Ni-rich) [24]	Per kg NCA-eq battery-grade material; industrial data	Mining 193.9; recycled EoL batteries 44.4 MJ	Mining 14.5; recycled EoL batteries 6.1 kg CO_2_-eq	—	GHG varies up to 5× with grid electricity
Ciez and Whitacre 2019 (NMC622, NCA, LFP) [6]	Attributional LCA + process cost models	—	Pyro/hydro: no significant reduction; direct: potential reduction; LFP: no net avoided emissions	Direct potentially competitive	Outputs as metal offsets, not cathode salts

**Table 6:** Qualitative decision matrix for the recycling route by cathode chemistry.

Cathode Chemistry	Pyrometallurgy	Hydrometallurgy	Direct Regeneration	Main Reason
LCO	Viable (high Co value)	Preferred	Viable for sorted consumer cells	Co value covers cost; feed often mixed
NMC111–532	As pretreatment only (reductive calcination)	Preferred for mixed feed	Preferred for sorted, moderately degraded feed	Degradation largely reversible; legacy compositions
NMC622–811, NCA	As pretreatment only	Preferred	Limited; mainly upcycling	Cracking, proton sensitivity, moving product specification
LFP	Not justified	Price-dependent; marginal economics	Preferred	Functional value exceeds elemental value; reversible defects
Mixed NMC + LFP black mass	Not justified for the LFP fraction	Default at present	Requires NMC/LFP separation first	Sorting is the bottleneck

**Table 7:** Minimum reporting set for cathode recycling studies, linked to the gates G1–G6 of the cathode-to-cathode framework.

No.	Item (Gate)	Quantity to Report	Unit	Minimum Acceptable Specification
1	Feed origin and ageing history (G1)	Source (production scrap, field-aged or laboratory-cycled cells); chemistry and grade; SOH distribution; ageing mode and storage history	–; % SOH	Chemistry confirmed analytically; SOH range and mean of the batch
2	Feed composition (G1–G2)	Cathode grade of the feed; graphite, Al, Cu, Fe, P and F contents; fraction of a second cathode chemistry	wt%	Measured on the actual feed; F and Al always reported for thermally pretreated feed
3	Pretreatment history (G2)	Discharge, comminution and binder-removal method; maximum temperature, atmosphere, duration	°C; h	Complete conditions, classified as in Table 2
4	Recovery yield (G3)	Mass of cathode material or precursor recovered per mass of cathode in the feed; element-specific recoveries	%	Closed mass balance; extraction and product yield reported separately
5	Process inputs (G3–G4)	Acid, reductant, Li source and Li excess relative to stoichiometry; water and wastewater	kg or mol per kg product; mol% excess	Normalised per kg of product, not only per kg of feed
6	Thermal budget (G3–G4)	Temperature, time and atmosphere of every thermal step	°C; h	All steps, including washing and tempering after molten-salt treatment
7	Product composition and structure (G4)	Li/TM ratio, cation mixing, phase purity, residual Al, Cu, Fe, F and C, particle size and morphology	–; %; ppm; µm	Compared with the specification of the intended commercial product
8	Benchmark (G5)	Reference material tested under the identical protocol	–	Commercial cathode of the same composition; pristine material from the same cell type as a minimum
9	Cell format and loading (G5)	Cell type, active-material loading, areal capacity, electrolyte amount	mg cm^−2^; mAh cm^−2^; g Ah^−1^	Full cell with graphite anode at practical areal capacity (≥2 mAh cm^−2^)
10	Durability (G5)	Capacity and rate capability; cycles to 80% retention; calendar (storage) ageing	mAh g^−1^; cycles; months	≥1000 full-cell cycles or cycling to 80% retention; storage test at elevated temperature and high SOC
11	Batch consistency (G4–G5)	Variation in composition, impurity content and capacity between production batches	coefficient of variation, %	At least three independent batches
12	Reuse (G6)	Fraction of recovered material incorporated into newly manufactured cells	%	Reported where available, with the qualification status of the material
13	Environmental and economic indicators	Energy, GHG emissions and cost per functional unit; boundary; grid mix; treatment of sorting	MJ kg^−1^; kg CO_2_-eq kg^−1^; $ kg^−1^	Conventions of Section 6.4

SOH, state of health; TM, transition metal; GHG, greenhouse gas.

**Table 8:** Illustrative application of the six-gate framework (Figure 7) to direct regeneration of LFP and hydrometallurgical resynthesis of Ni-rich layered oxides.

Gate	Case A: Direct Regeneration of LFP (Xu et al. [14])	Case B: Hydrometallurgical Resynthesis of Ni-rich NMC/NCA [24,49,50,51,54]
G1 Identification	Single-chemistry LFP cells with known SOH (40–85%) [14]. Met for laboratory feed; chemistry identification of industrial feed not demonstrated.	Mixed Ni-rich feed is accepted; composition is adjusted in solution [24]. Met at industrial scale.
G2 Separation	Cathode powder harvested from dismantled cells with NMP [14]. Partly met: purity specification for industrial LFP black mass not established (Section 3.3, Section 3.4 and Section 3.5).	Impurities are removed in solution; tolerance windows are defined for Al and C in NMC622 (0.2 at% acceptable, 5 at% detrimental) [49,50,51]. Met.
G3 Recovery	Mass yield of regenerated LFP per cathode input not reported. Not met (not reported).	Recovery to battery-grade mixed sulphates at industrial scale [24]. Met for metal recovery; product yield per cathode input should be stated.
G4 Restoration	Olivine phase restored; anti-site defects 4.81% → 1.5% (pristine 2.5%) [14]. Met.	Resynthesised NMC111 meets commercial specifications [54]; impurity windows for Ni-rich grades are composition specific [49,50,51]. Met for NMC111, partly met for Ni-rich grades.
G5 Validation	Electrodes at 19 mg cm^−2^ in graphite pouch cells; 150 mAh g^−1^ after 1000 cycles; benchmarked against fresh cells [14]. Met at laboratory scale.	1 Ah cells: 4200 cycles to 80% retention, 33% longer than the commercial control, but for NMC111 [54]; equivalent Ah-scale validation of resynthesised Ni-rich NMC not identified in this review. Partly met.
G6 Reuse	Not demonstrated. Not met.	Battery-grade intermediates produced industrially [24]; fraction entering new cells not reported. Partly met.
Limiting gates	G1–G3 and G6: feed identification, separation and unreported yield	G5 and G6 for Ni-rich grades: Ah-scale validation and reported reuse

## Data Availability

No new data were created or analysed in this study.

## Abbreviations

The following abbreviations are used in this manuscript:

DFT	density functional theory
EoL	end of life
EV	electric vehicle
GHG	greenhouse gas
HF	hydrogen fluoride
LCA	life-cycle assessment
LCO	LiCoO_2_
LFP	LiFePO_4_
Li_2_DHBN	3,4-dihydroxybenzonitrile dilithium
LIB	lithium-ion battery
LMFP	LiMn_x_Fe_1−x_PO_4_
LMO	LiMn_2_O_4_
MIBC	methyl isobutyl carbinol
NCA	LiNi_0.8_Co_0.15_Al_0.05_O_2_
NMC	LiNixMnyCozO2; NMC is used throughout, including for specific grades (e.g., NMC523); the alternative notation NCM is retained only in the titles of cited works
NMP	N-methyl-2-pyrrolidone
n.r.	not reported
PVDF	polyvinylidene fluoride
SC	single-crystal
SEI	solid–electrolyte interphase
SOH	state of health
TEA	techno-economic analysis
TM	transition metal

## References

[1] International Energy Agency (2025). Global Critical Minerals Outlook 2025.

[2] Lombardo T., Paoli L., Fernandez Pales A., Gül T. (2026). Global Battery Markets Are Growing Strongly—And So Are the Supply Risks (Commentary).

[3] International Energy Agency (2026). Electric Vehicle Batteries. Global EV Outlook 2026.

[4] Huang B., Pan Z., Su X., An L. (2018). Recycling of Lithium-Ion Batteries: Recent Advances and Perspectives. J. Power Sources.

[5] Olivetti E.A., Ceder G., Gaustad G.G., Fu X. (2017). Lithium-Ion Battery Supply Chain Considerations: Analysis of Potential Bottlenecks in Critical Metals. Joule.

[6] Ciez R.E., Whitacre J.F. (2019). Examining Different Recycling Processes for Lithium-Ion Batteries. Nat. Sustain..

[7] (2023). Regulation (EU) 2023/1542 of the European Parliament and of the Council of 12 July 2023 Concerning Batteries and Waste Batteries, Amending Directive 2008/98/EC and Regulation (EU) 2019/1020 and Repealing Directive 2006/66/EC. Off. J. Eur. Union.

[8] Zou Y., Chernyaev A., Ossama M., Seisko S., Lundström M. (2024). Leaching of NMC Industrial Black Mass in the Presence of LFP. Sci. Rep..

[9] Harper G., Sommerville R., Kendrick E., Driscoll L., Slater P., Stolkin R., Walton A., Christensen P., Heidrich O., Lambert S. (2019). Recycling Lithium-Ion Batteries from Electric Vehicles. Nature.

[10] Fan E., Li L., Wang Z., Lin J., Huang Y., Yao Y., Chen R., Wu F. (2020). Sustainable Recycling Technology for Li-Ion Batteries and Beyond: Challenges and Future Prospects. Chem. Rev..

[11] Xiao J., Li J., Xu Z. (2020). Challenges to Future Development of Spent Lithium Ion Batteries Recovery from Environmental and Technological Perspectives. Environ. Sci. Technol..

[12] Li L., Zhang X., Li M., Chen R., Wu F., Amine K., Lu J. (2018). The Recycling of Spent Lithium-Ion Batteries: A Review of Current Processes and Technologies. Electrochem. Energy Rev..

[13] Ji G., Wang J., Liang Z., Jia K., Ma J., Zhuang Z., Zhou G., Cheng H.-M. (2023). Direct Regeneration of Degraded Lithium-Ion Battery Cathodes with a Multifunctional Organic Lithium Salt. Nat. Commun..

[14] Xu P., Dai Q., Gao H., Liu H., Zhang M., Li M., Chen Y., An K., Meng Y.S., Liu P. (2020). Efficient Direct Recycling of Lithium-Ion Battery Cathodes by Targeted Healing. Joule.

[15] Li J., Du Z., Ruther R.E., An S.J., David L.A., Hays K., Wood M., Phillip N.D., Sheng Y., Mao C. (2017). Toward Low-Cost, High-Energy Density, and High-Power Density Lithium-Ion Batteries. JOM.

[16] Ordoñez J., Gago E.J., Girard A. (2016). Processes and Technologies for the Recycling and Recovery of Spent Lithium-Ion Batteries. Renew. Sustain. Energy Rev..

[17] Cusenza M.A., Bobba S., Ardente F., Cellura M., Di Persio F. (2019). Energy and Environmental Assessment of a Traction Lithium-Ion Battery Pack for Plug-In Hybrid Electric Vehicles. J. Clean. Prod..

[18] Meshram P., Mishra A., Abhilash, Sahu R. (2020). Environmental Impact of Spent Lithium Ion Batteries and Green Recycling Perspectives by Organic Acids—A Review. Chemosphere.

[19] Ryu H.-H., Park K.-J., Yoon C.S., Sun Y.-K. (2018). Capacity Fading of Ni-Rich Li[Ni_x_Co_y_Mn_1–x–y_]O_2_ (0.6 ≤ x ≤ 0.95) Cathodes for High-Energy-Density Lithium-Ion Batteries: Bulk or Surface Degradation?. Chem. Mater..

[20] Ryu H.-H., Park G.-T., Yoon C.S., Sun Y.-K. (2018). Microstructural Degradation of Ni-Rich Li[Ni_x_Co_y_Mn_1−x−y_]O_2_ Cathodes during Accelerated Calendar Aging. Small.

[21] He F., Lv Y., Wu J., Zhang Q., Hao S., Yuan L., Yang H., Yang Y. (2025). Molten Salt Regeneration of Single-Crystal LiNi_0.8_Co_0.1_Mn_0.1_O_2_ from End-of-Life Cathodes. Energy Environ. Nexus.

[22] Shi Y., Zhang M., Meng Y.S., Chen Z. (2019). Ambient-Pressure Relithiation of Degraded Li_x_Ni_0.5_Co_0.2_Mn_0.3_O_2_ (0 < x < 1) via Eutectic Solutions for Direct Regeneration of Lithium-Ion Battery Cathodes. Adv. Energy Mater..

[23] Liu S., Bokov A., Trouillet V., Zimmermanns R., Indris S., Nandy S., Mereacre L., Missyul A., Bergfeldt T., Knapp M. (2026). Effect of Impurities on Direct Recycling of Spent LiNi_x_Co_y_Mn_z_O_2_ Cathode Material for Lithium-Ion Batteries. ACS Nano.

[24] Machala M.L., Chen X., Bunke S.P., Forbes G., Yegizbay A., de Chalendar J.A., Azevedo I.L., Benson S., Tarpeh W.A. (2025). Life Cycle Comparison of Industrial-Scale Lithium-Ion Battery Recycling and Mining Supply Chains. Nat. Commun..

[25] Li Y., Ye Z., Wang S., Zhong H., Cao Z., Ma X. (2026). Direct Regeneration of Cathodes from Spent LIB Black Mass through an Integrated Roasting-Flotation Method with Impurity-Tailored Self-Decontamination. J. Mater. Chem. A.

[26] Salces A.M., Henderson M.S., Rodríguez-Medina A.J., Bachmann K., Oraby E., Beh C.C., Rudolph M., Eksteen J., Vanderbruggen A. (2025). Evaluation of a Bio-Based Solvent Pretreatment for Sustainable Froth Flotation of Black Mass from Spent Lithium-Ion Batteries. ACS Sustain. Resour. Manag..

[27] Vanderbruggen A., Salces A., Ferreira A., Rudolph M., Serna-Guerrero R. (2022). Improving Separation Efficiency in End-of-Life Lithium-Ion Batteries Flotation Using Attrition Pre-Treatment. Minerals.

[28] Zhang J., Li J., Wang Y., Sun M., Wang L., Tu Y. (2023). Separation of Graphites and Cathode Materials from Spent Lithium-Ion Batteries Using Roasting–Froth Flotation. Sustainability.

[29] Renier O., Pellini A., Spooren J. (2023). Advances in the Separation of Graphite from Lithium Iron Phosphate from End-of-Life Batteries Shredded Fine Fraction Using Simple Froth Flotation. Batteries.

[30] Liu X., Bu X., Ni C., Ping A., Wu F., Xie G., Ulusoy U. (2026). Assessment of the Flotation Separation of Four Typical LIB Cathodes: Emphasizing the Unique Flotation Behavior Propelled by the Combined Impact of Ultrafine Particle Size and LiFePO_4_ Surface Properties. Sep. Purif. Technol..

[31] Tasawar A., Munchen D.D., Birich A., Yeetsorn R., Friedrich B. (2025). Effect of Oxidative Roasting on Selective Leaching of Lithium from Industrially Shredded Lithium Iron Phosphate Blackmass. Metals.

[32] Oboh R., Huang K., Pan L. (2025). Overcoming Heterocoagulation Challenge in Selective Flotation Separation between Lithium Cobalt Oxide and Lithium Manganese Oxide. Sep. Purif. Technol..

[33] Henderson M.S., Beh C.C., Oraby E., Eksteen J. (2025). Organic Acid Leaching of Black Mass with an LFP and NMC Mixed Chemistry. Recycling.

[34] Rothermel S., Evertz M., Kasnatscheew J., Qi X., Grützke M., Winter M., Nowak S. (2016). Graphite Recycling from Spent Lithium-Ion Batteries. ChemSusChem.

[35] Gupta V., Potts C., Tran D., Gao H., Li M., Lin J., Chen Z. (2025). Streamlining Ni-Rich LiNi_x_Mn_y_Co_z_O_2_ Cathode Black Mass Purification for Direct Recycling and Upcycling through the Alkoxythermal Process. Adv. Energy Mater..

[36] Thompson D.L., Hartley J.M., Lambert S.M., Shiref M., Harper G.D.J., Kendrick E., Anderson P., Ryder K.S., Gaines L., Abbott A.P. (2020). The Importance of Design in Lithium Ion Battery Recycling—A Critical Review. Green Chem..

[37] Georgi-Maschler T., Friedrich B., Weyhe R., Heegn H., Rutz M. (2012). Development of a Recycling Process for Li-Ion Batteries. J. Power Sources.

[38] Bae H., Kim Y. (2021). Technologies of Lithium Recycling from Waste Lithium Ion Batteries: A Review. Mater. Adv..

[39] Wang R.-C., Lin Y.-C., Wu S.-H. (2009). A Novel Recovery Process of Metal Values from the Cathode Active Materials of the Lithium-Ion Secondary Batteries. Hydrometallurgy.

[40] Chen W.S., Ho H.J. (2018). Leaching Behavior Analysis of Valuable Metals from Lithium-Ion Batteries Cathode Material. Key Eng. Mater..

[41] He L.-P., Sun S.-Y., Mu Y.-Y., Song X.-F., Yu J.-G. (2017). Recovery of Lithium, Nickel, Cobalt, and Manganese from Spent Lithium-Ion Batteries Using L-Tartaric Acid as a Leachant. ACS Sustain. Chem. Eng..

[42] Meshram P., Pandey B.D., Mankhand T.R. (2015). Recovery of Valuable Metals from Cathodic Active Material of Spent Lithium Ion Batteries: Leaching and Kinetic Aspects. Waste Manag..

[43] Zeng X., Li J., Shen B. (2015). Novel Approach to Recover Cobalt and Lithium from Spent Lithium-Ion Battery Using Oxalic Acid. J. Hazard. Mater..

[44] Li L., Ge J., Chen R., Wu F., Chen S., Zhang X. (2010). Environmental Friendly Leaching Reagent for Cobalt and Lithium Recovery from Spent Lithium-Ion Batteries. Waste Manag..

[45] Jha M.K., Kumari A., Jha A.K., Kumar V., Hait J., Pandey B.D. (2013). Recovery of Lithium and Cobalt from Waste Lithium Ion Batteries of Mobile Phone. Waste Manag..

[46] Chen X., Fan B., Xu L., Zhou T., Kong J. (2016). An Atom-Economic Process for the Recovery of High Value-Added Metals from Spent Lithium-Ion Batteries. J. Clean. Prod..

[47] Li L., Ge J., Wu F., Chen R., Chen S., Wu B. (2010). Recovery of Cobalt and Lithium from Spent Lithium Ion Batteries Using Organic Citric Acid as Leachant. J. Hazard. Mater..

[48] Joulié M., Laucournet R., Billy E. (2014). Hydrometallurgical Process for the Recovery of High Value Metals from Spent Lithium Nickel Cobalt Aluminum Oxide Based Lithium-Ion Batteries. J. Power Sources.

[49] Zhang R., Zheng Y., Yao Z., Vanaphuti P., Ma X., Bong S., Chen M., Liu Y., Cheng F., Yang Z. (2020). Systematic Study of Al Impurity for NCM622 Cathode Materials. ACS Sustain. Chem. Eng..

[50] Zheng Y., Zhang R., Vanaphuti P., Fu J., Yang Z., Wang Y. (2021). Unveiling the Influence of Carbon Impurity on Recovered NCM622 Cathode Material. ACS Sustain. Chem. Eng..

[51] Meng Z., Ma X., Hou J., Zheng Y., Wang Y. (2025). Impurity Impacts of Recycling NMC Cathodes. Adv. Energy Mater..

[52] Yang Y., Xu S., He Y. (2017). Lithium Recycling and Cathode Material Regeneration from Acid Leach Liquor of Spent Lithium-Ion Battery via Facile Co-Extraction and Co-Precipitation Processes. Waste Manag..

[53] Gao W., Zhang X., Zheng X., Lin X., Cao H., Zhang Y., Sun Z. (2017). Lithium Carbonate Recovery from Cathode Scrap of Spent Lithium-Ion Battery: A Closed-Loop Process. Environ. Sci. Technol..

[54] Ma X., Chen M., Zheng Z., Bullen D., Wang J., Harrison C., Gratz E., Lin Y., Yang Z., Zhang Y. (2021). Recycled Cathode Materials Enabled Superior Performance for Lithium-Ion Batteries. Joule.

[55] Bian D., Sun Y., Li S., Tian Y., Yang Z., Fan X., Zhang W. (2016). A Novel Process to Recycle Spent LiFePO_4_ for Synthesizing LiFePO_4_/C Hierarchical Microflowers. Electrochim. Acta.

[56] Lan Y., Li X., Zhou G., Yao W., Cheng H.-M., Tang Y. (2024). Direct Regenerating Cathode Materials from Spent Lithium-Ion Batteries. Adv. Sci..

[57] Chen J., Li Q., Song J., Song D., Zhang L., Shi X. (2016). Environmentally Friendly Recycling and Effective Repairing of Cathode Powders from Spent LiFePO_4_ Batteries. Green Chem..

[58] Shi Y., Chen G., Liu F., Yue X., Chen Z. (2018). Resolving the Compositional and Structural Defects of Degraded LiNi_x_Co_y_Mn_z_O_2_ Particles to Directly Regenerate High-Performance Lithium-Ion Battery Cathodes. ACS Energy Lett..

[59] Xu P., Guo X., Jiao B., Chen J., Zhang M., Liu H., Yu X., Appleberry M., Yang Z., Gao H. (2024). Proton-Exchange Induced Reactivity in Layered Oxides for Lithium-Ion Batteries. Nat. Commun..

[60] Jiang G., Zhang Y., Meng Q., Zhang Y., Dong P., Zhang M., Yang X. (2020). Direct Regeneration of LiNi_0.5_Co_0.2_Mn_0.3_O_2_ Cathode from Spent Lithium-Ion Batteries by the Molten Salts Method. ACS Sustain. Chem. Eng..

[61] Qin Z., Wen Z., Xu Y., Zheng Z., Bai M., Zhang N., Jia C., Wu H.B., Chen G. (2022). A Ternary Molten Salt Approach for Direct Regeneration of LiNi_0.5_Co_0.2_Mn_0.3_O_2_ Cathode. Small.

[62] Ma J., Wang J., Jia K., Liang Z., Ji G., Zhuang Z., Zhou G., Cheng H.-M. (2022). Adaptable Eutectic Salt for the Direct Recycling of Highly Degraded Layer Cathodes. J. Am. Chem. Soc..

[63] Liu X., Wang R., Liu S., Pu J., Xie H., Wu M., Liu D., Li Y., Liu J. (2023). Organic Eutectic Salts-Assisted Direct Lithium Regeneration for Extremely Low State of Health Ni-Rich Cathodes. Adv. Energy Mater..

[64] Wang T., Luo H., Bai Y., Li J., Belharouak I., Dai S. (2020). Direct Recycling of Spent NCM Cathodes through Ionothermal Lithiation. Adv. Energy Mater..

[65] Mehboob G., Yuan B., Ahmad A., Shah B.A., Mehboob G., Lin W., Ajmal S., Peng W., Yang G., Liu J. (2025). Direct Regeneration of Highly Degraded Cathode Materials from Spent Lithium-Ion Batteries via Eutectic Molten Salts with CoO and MnO_2_ Additives. Chem. Eng. J..

[66] Fan M., Meng Q., Chang X., Gu C.-F., Meng X.-H., Yin Y.-X., Li H., Wan L.-J., Guo Y.-G. (2022). In Situ Electrochemical Regeneration of Degraded LiFePO_4_ Electrode with Functionalized Prelithiation Separator. Adv. Energy Mater..

[67] Zhou H., Ge P., Li J., Zeng Z., Wang B., Dong Z., Yang Y. (2025). Homogenizing Phase–Component Regeneration of Spent NCM811 into Single-Crystal NCM622 with Enhanced Structural and Electrochemical Stability. Energy Storage Mater..

[68] Shao Y., Hao S., Liu F., Kong Y., Ji H., Xie Z., Liao Y., Liu W., Huang Y., Yuan L. (2026). Direct Regeneration of Spent LiNi_0.8_Co_0.1_Mn_0.1_O_2_ into Ce-Doped Single-Crystal Cathodes. Chem. Bio Eng..

[69] Qin Z., Zhang Y., Luo W., Zhang T., Wang T., Ni L., Wang H., Zhang N., Liu X., Zhou J. (2023). A Universal Molten Salt Method for Direct Upcycling of Spent Ni-Rich Cathode towards Single-Crystalline Li-Rich Cathode. Angew. Chem. Int. Ed..

[70] Ji G., Zheng N., Chen Y., Ji H., Wang J., Li H., Liang Z., Cheng H.-M., Zhou G. (2026). All-Dry and Scalable Direct Recycling of Spent Ternary Black Mass toward Long-Life Ah-Level Pouch Cell. Adv. Mater..

[71] Masias A., Marcicki J., Paxton W.A. (2021). Opportunities and Challenges of Lithium Ion Batteries in Automotive Applications. ACS Energy Lett..

[72] Rieger J., Stuhr S., Rutrecht B., Morgenbesser S., Nigl T., Arnberger A., Kunanz H., Lesiak S. (2025). Graphite Separation from Lithium-Ion Battery Black Mass Using Froth Flotation and Quality Evaluation for Reuse as a Secondary Raw Material Including Non-Battery Applications. Recycling.

[73] Tian H., Graczyk-Zajac M., Kessler A., Weidenkaff A., Riedel R. (2024). Recycling and Reusing of Graphite from Retired Lithium-Ion Batteries: A Review. Adv. Mater..

[74] Ji S., Zhang A., Hua W., Yan S., Chen X. (2024). Regeneration of Graphite from Spent Lithium-Ion Batteries as Anode Materials through Stepwise Purification and Mild Temperature Restoration. Battery Energy.

[75] Liu G., Ma L., Xi X., Nie Z. (2024). Efficient Purification and High-Quality Regeneration of Graphite from Spent Lithium-Ion Batteries by Surfactant-Assisted Methanesulfonic Acid. Waste Manag..

[76] Kosenko A., Pushnitsa K., Chernyavsky V., Novikov P., Popovich A.A. (2024). Graphite Regeneration and NCM Cathode Type Synthesis from Retired LIBs by Closed-Loop Cycle Recycling Technology of Lithium-Ion Batteries. Energies.

[77] Yu R., Zhou C., Zhou X., Yang J., Tang J., Zhang Y. (2024). Efficient Regeneration of Graphite from Spent Lithium-Ion Batteries through Combination of Thermal and Wet Metallurgical Approaches. Materials.

[78] Li G., Chen C., Chen Z., Zheng J., Kang Z., Cheng H., Li X., Yu H., Tian Z., Lai Y. (2026). Closed-Loop Graphite Recycling from the Manufacturing Scrap Graphite by Sulfuric Acid Leaching Combined with Low-Temperature Oxidation. Waste Manag..

[79] (2006). Environmental Management—Life Cycle Assessment—Requirements and Guidelines.

[80] Bachér J., Jenu S., Kajolinna T. (2025). Identification of Waste Lithium-Ion Battery Cell Chemistry for Recycling. Waste Manag..

